# Nanozyme-Powered Multimodal Sensing for Pesticide Detection

**DOI:** 10.3390/foods14111957

**Published:** 2025-05-30

**Authors:** Binfeng Yin, Zhuoao Jiang, Rashid Muhammad, Jun Liu, Junjie Wang

**Affiliations:** 1 School of Mechanical Engineering, Yangzhou University, Yangzhou 225127, China; jza2422443660@outlook.com (Z.J.); chemistwindow@gmail.com (R.M.); junjiewang0803@foxmail.com (J.W.); 2 Suqian Product Quality Supervision and Inspection Institute, Suqian 223800, China; ljljljlj1982@163.com

**Keywords:** pesticide residues, nanozymes, multimodal sensing, AI, food safety

## Abstract

The detection of pesticide residues in food is crucial for ensuring food safety, safeguarding public health, and promoting sustainable development. Overusing pesticides on agricultural crops can lead to the emergence of various diseases. Traditional methods for detecting pesticides offer high precision with limitations like high cost, the requirement of expert technicians, and tedious analytical procedures. To address these issues, nanozymes have been widely applied due to their advantages such as low cost, high stability, and high sensitivity. This review summarizes the research progress of nanozymes in the detection of pesticide residues in food over the last decade, focusing on the synthesis strategies and catalytic mechanisms of carbon-based, metal-based, metal-oxide-based, metal–organic framework (MOF)-based, fluorescence-based, and other X-based nanozymes. This review covers the application of multimodal sensing based on nanozymes in the detection of pesticides, including colorimetric/fluorescence, fluorescence/photothermal, photothermal/colorimetric, and other multimodal sensing techniques. Finally, this review discusses the main challenges currently faced by nanozymes in the detection of pesticides and the current applications of using AI with nanozymes. It also presents future development prospects, with the aim of providing references for the selection of X-based nanozymes and the choice of appropriate detection methods when dealing with traditional and new pesticides in combination with AI.

## 1. Introduction

As a core tool in modern agricultural production [1,2], pesticides play an irreplaceable role in controlling diseases [3,4], pests [5,6], and weeds [7,8], regulating crop physiological metabolism and ensuring food security [9,10,11]. According to the Food and Agriculture Organization of the United Nations, pests and diseases can increase food losses by 30% to 40% annually [12]. These losses are mainly controlled by using synthetic pesticides [13]. The Resistance Action Committees on insecticides (IRAC), herbicides (HRAC) and fungicides (FRAC) have classified these pesticides based on their mode of action. Among these, pesticides from organophosphate, pyrethroids, carbamates, sulfonylureas, triazoles, and some other classes are commonly used in Integrated Pest Management (IPM) programs. Due to the illegitimate use of pesticides, these pesticides accumulate and concentrate either in agricultural products or in the environment [14,15], ultimately causing serious pollution in the form of pesticide residues in the ecological environment and agricultural products. Pesticide residues in food have become a global public health and safety issue [16,17,18]. Excessive pesticide residues in food may cause acute poisoning [19], such as nausea [20], vomiting [21], and breathing difficulties [22], and long-term exposure may lead to chronic harm [23], such as nervous system damage [24], endocrine disorders, and even cancer [25], especially for sensitive groups such as children and pregnant women [26,27]. Although the use of pesticides has decreased in recent years, food safety incidents caused by the illegal use of pesticides still occur every year [28,29,30]. Different regulatory agencies such as Codex Alimentarius and the EU have established different maximum residue limits (MRLs) for these unwanted residues in food. When pesticide residues in food exceed these limits, the food is considered unfit for human consumption. According to the Rapid Alert System for Food and Feed (RASFF) import alert database, from January 2025 to April 2025, more than 300 consignments of agricultural commodities were found to be contaminated with different pesticides, in which chlorpyrifos, acetamiprid, imidacloprid, thiamethoxam, clothianidin, chlorfenapyr carbendazim, and some others were reported multiple times. Therefore, the rapid and accurate detection of pesticide residues in food is of great significance [31,32,33,34,35].

Traditional methods used for detecting pesticide residues in food mainly include spectroscopic methods [36,37,38]. In traditional analytical methods, the pesticide residues are first extracted from samples using different extraction and cleanup techniques such as Solid-Phase Extraction (SPE) or the QuEChERS method [39,40,41,42]. Then, the samples are analyzed on GC-MS/MS or LC-MS/MS for quantification [42,43,44,45]. Although these methods have high sensitivity and accuracy [46,47], they are highly dependent on equipment [48,49], costly [50,51], tedious [52], and have poor timeliness [53,54].

With the development of nanotechnology [55], nanozymes, as nanomaterials with biomimetic catalytic activity [56,57], have shown great potential in food pesticide residue detection [58,59,60]. The catalytic activity of nanozymes can be significantly enhanced by developing different structures and surface modifications [61]. In recent years, a large number of biosensors based on nanozymes have been proposed and applied in pesticide residue detection [62,63,64], mainly including carbon-based [63,65], metal-based [65,66], metal-oxide-based [67,68], metal–organic framework-based [69,70], fluorescence-based [71,72], and other composite material-based nanozymes [73,74]. Their catalytic mechanisms cover various types of enzyme activities such as oxidoreductases and hydrolases [75]. Compared with biosensors relying on natural enzymes, nanozyme sensors have overcome the environmental sensitivity of natural enzymes and can maintain stable catalytic performance under extreme pH or temperature conditions [76,77,78]. Specific recognition of pesticides can be achieved through aptamer modification or antibody coupling [79]. Combined with microfluidic chips or paper-based detection technologies [80,81], portable detection devices can be constructed, increasing the detection sensitivity by 2–3 orders of magnitude while reducing the cost by approximately 80%, making them powerful tools for the rapid and accurate detection of food pesticide residues [82].

**Scheme 1:**
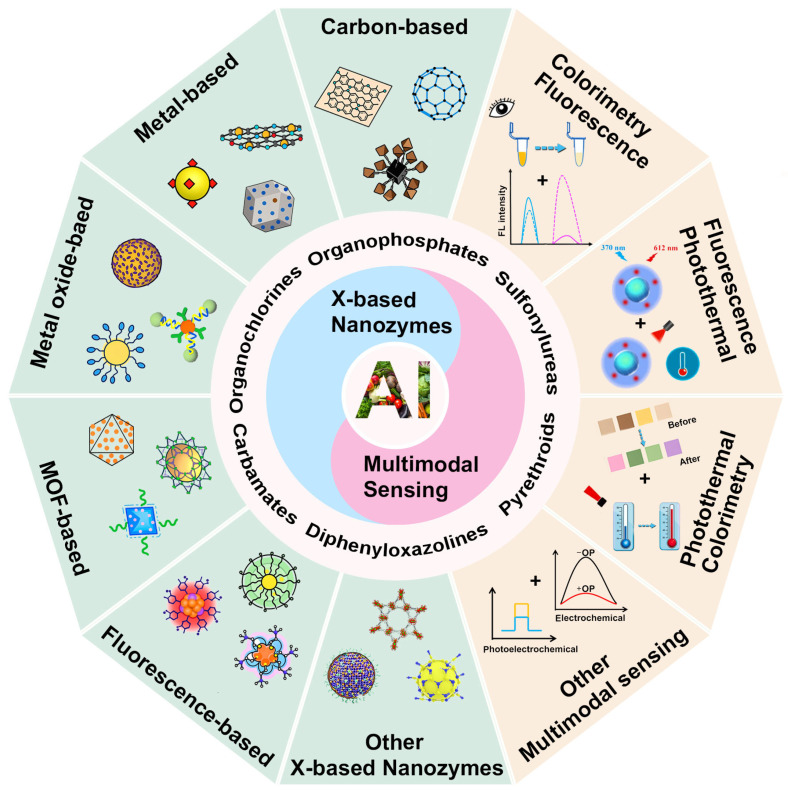
X-based nanozymes and multimodal sensing for food safety analysis.

Currently, most review papers on nanozymes in the field of pesticide detection focus on the technical application aspect, mainly discussing the catalytic mechanism or sensing mechanism of nanozymes [83,84,85]. However, the design of nanozymes on different types of substrates and the systematic analysis of their selectivity for different pesticides still need to be elaborated [86]. In addition, multimodal pesticide detection based on nanozymes, which significantly increases the detection sensitivity by mutual verification of multiple signals, is also a current trend in research [87]. No relevant introduction or summary is available in other reviews on nanozymes. In this regard, this review mainly introduces the synthesis methods and catalytic mechanisms of nanozymes on different substrates, including carbon-based, metal-based, metal-oxide-based, metal–organic framework-based, fluorescence-based, and other composite material-based nanozymes. Then, it introduces the application of nanozyme-driven multimodal sensing technology in pesticide detection, including colorimetric/fluorescence, fluorescence/photothermal, photothermal/colorimetric and other multimodal sensing technologies, as illustrated in Scheme 1. Finally, this paper summarizes the challenges faced by nanozymes in food pesticide residue detection and the current application status of “AI + nanozymes”, and looks forward to future development. Our aim is to provide researchers with references for selecting different substrate-based nanozymes and appropriate sensing technologies when facing traditional and new pesticides in the future, by combining AI to selectively construct nanozymes on different substrates.

## 2. X-Based Nanozymes

Due to their advantages of high catalytic activity and environmental stability, nanozymes have become the core tools for the rapid on-site detection of pesticide residues in food [88,89]. Nanozymes are classified into carbon-based nanozymes, metal-based nanozymes, metal-oxide-based nanozymes, MOF-based nanozymes, and other X-based nanozymes (nanozymes based on other composite materials). Each type of nanozyme has special characteristics [90], synthesis [91], and catalytic mechanisms [92], and the applications of these nanozymes for the identification and quantification of pesticide residues in food are elaborated on below. Table 1 lists X-based nanozymes used for pesticide detection.

### 2.1. Carbon-Based Nanozymes

Carbon-based nanozymes are a class of nanomaterials with catalytic activity similar to enzymes, which are supported by carbon materials [130]. Various carbon-based nanozymes have been developed such as graphene [131], carbon nanotubes [132], carbon dots [133,134], fullerenes [135], or carbon nanospheres [136]. The catalytic activity of these nanozymes depends on the surface functional groups and dopant elements [137]. For example, oxygen-containing functional groups, such as hydroxyl and carbonyl, combine with substrates through hydrogen bonding [138]. The doping of elements enhances catalytic activity [139]. The sp^2^-hybridized carbon materials, and double bonds (π-π conjugation) in graphene, can provide electron transfer channels and stabilize intermediate products [140,141]. Carbon-based nanozymes can be further categorized into pure carbon-based nanozymes, carbon-based nanozymes doped with non-metallic atoms, carbon-based nanozymes doped with metallic atoms, and carbon-based nanozymes doped with monatomic metals.

#### 2.1.1. Pure Carbon-Based Nanozymes

Pure carbon-based nanozymes can undergo catalytic activity without any metal atoms [142]. They are stable at high temperatures or in acidic conditions. These nanozymes are suitable for the detection of pesticide residues in various food samples. For instance, Chonticha et al. synthesized graphene quantum dots (GQDs) for dichlorvos and methoxychlor analysis [93]. These GDQs were prepared by citric acid pyrolysis and then immobilized with acetylcholinesterase (AChE) and choline oxidase (CHOx) through enzyme immobilization technology to construct a GQD/AChE/CHOx nanozyme biosensor. The GQDs exhibit peroxidase-like activity due to the abundance of -COOH and -OH functional groups on their surface, which enables GQD nanozymes to have highly efficient catalytic properties. In the cascade enzymatic reaction of AChE and CHOx, acetylcholine is hydrolyzed to generate H_2_O_2_. The surface functional groups of GQDs act as active sites, facilitating the transfer of electrons from GQDs to the lowest unoccupied molecular orbital of H_2_O_2_, which triggers fluorescence quenching and results in the weakening of their own fluorescence. The limit of detection (LOD) for dichlorvos is 0.778 μM.

The principle of static quenching is based on the formation of non-fluorescent complexes between fluorescent molecules and quenchers in the ground state, which prevents light emission upon excitation. False-positive results arise due to the presence of interfering substances that bind with the fluorescent molecules to form non-specific complexes. False-negative results occur when the concentration of the target analyte is high, leading to excessive consumption of the quencher or saturation of the complex. In contrast, dynamic quenching takes place in the excited state of the fluorescent substance and relies on interactions through molecular collisions or energy transfer, rather than stable complex formation. This mechanism effectively circumvents the influence of non-specific binding, thereby avoiding the false-positive and false-negative issues associated with static quenching. Therefore, Li et al. developed a dynamic quenching mechanism for paraoxon [94]. This team used folate and p-phenylenediamine as precursors and carried out a hydrothermal reaction under alkaline conditions at 170 °C for 12 h. They successfully prepared carbon quantum dots with stable luminescence properties. In the presence of AChE, acetylthiocholine (ATCh) is catalyzed to produce thiocholine (TCh), which reacts with 5,5′-dithiobis (2-nitrobenzoic acid) (DTNB) to generate thiocholine-5-thio-2-nitrobenzoate (TNBA). The electrostatic attraction between positively charged TNBA and negatively charged CDs brings them closer, increasing the collision frequency and thereby shortening the fluorescence lifetime of CDs, which enhances the efficiency of dynamic quenching. When OPs are present, the activity of AChE is inhibited, leading to reduced production of TCh and TNBA. This results in the recovery of the fluorescence signal. Conversely, in the absence of OPs, the fluorescence signal is quenched. Based on the dual catalytic effect of AChE, the detection limit for paraoxon was 0.4 ng/mL. The recovery rate in water, rice, and cabbage detections was 90–102%, which was consistent with the results of gas chromatography.

To reduce the production costs associated with these nanozymes and make environmentally friendly nanozymes, Yue et al. prepared biochar nanozymes by pyrolysis of algal biomass [95], providing a new strategy for the resource utilization of agricultural wastes and the efficient detection of food pesticide residues. This team used Spirulina, Chlorella, and Enteromorpha as raw materials and subjected them to pyrolysis at 700 °C in argon gas for 3 h to obtain biochar materials: Spirulina biochar (SP-BC), Chlorella biochar (CH-BC), and Enteromorpha biochar (EP-BC). These biochars possess abundant surface functional groups and a graphitic structure. For example, structures such as -COOH, C-O, and aromatic carbons can mimic natural peroxidases. Carboxyl- and oxygen-containing functional groups attract the 3,3′,5,5′-tetramethylbenzidine (TMB) substrate through electrostatic interactions and reduce the activation energy of the reaction. The conjugated system of the aromatic carbon framework further accelerates electron transfer, forming a synergistic catalytic effect that catalyzes the colorimetric reaction of the TMB-H_2_O_2_ system. The detection limit for diafenthiuron, bensulfuron methyl, fomesafen, lactofen, and starane is as low as 1 μM, and they have been successfully applied to soil, lake water, seawater, and fruit and vegetable samples such as apples and cucumbers.

#### 2.1.2. Carbon-Based Nanozymes Doped with Other Atoms

**Figure 1:**
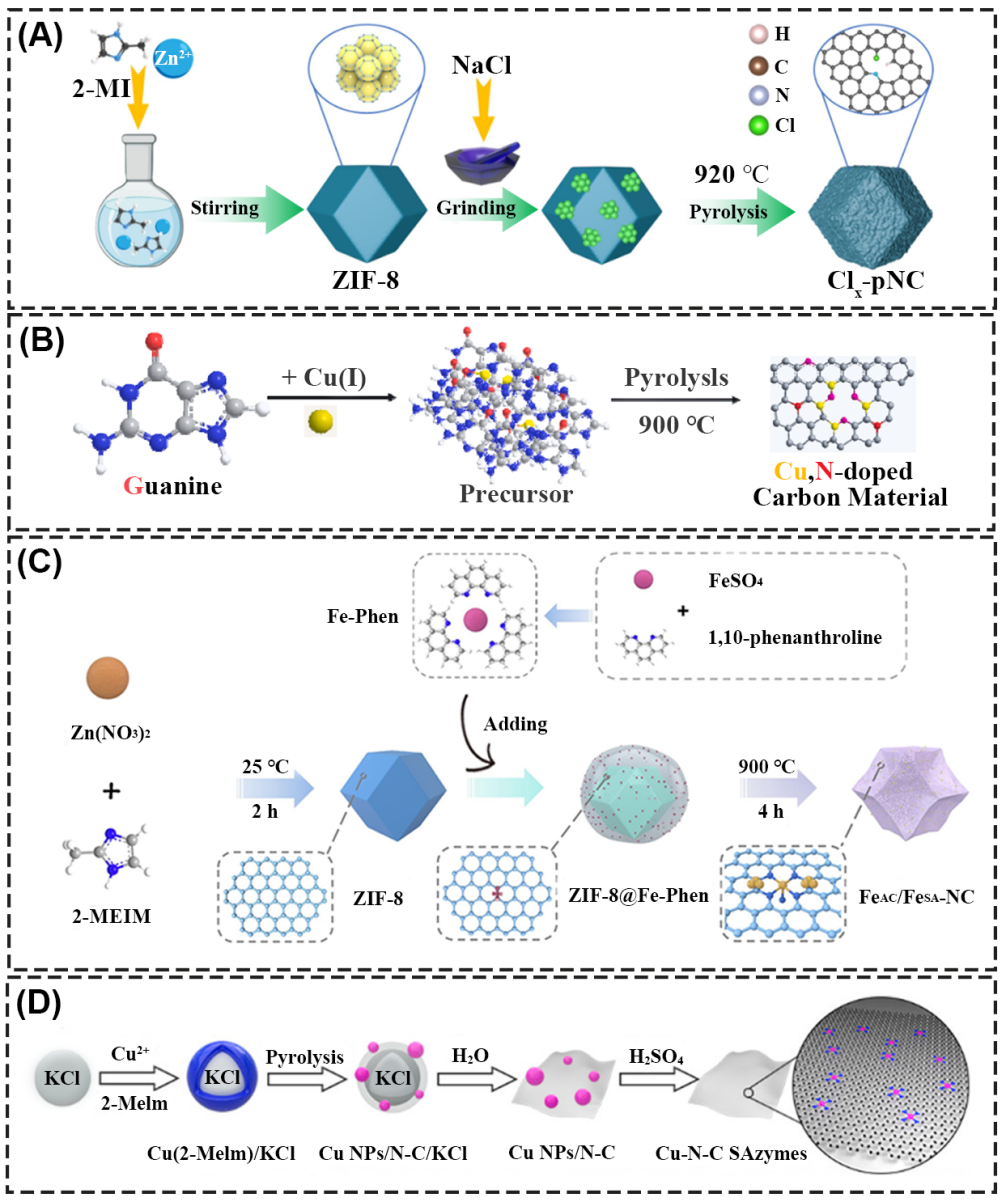
Schematic diagram of carbon-based nanozyme synthesis. (**A**) Cl_x_-pNC. (**B**) Cu@NC. (**C**) Fe_AC_/Fe_SA_-NC. (**D**) Cu-N-C.

Many carbon-based nanozymes are also doped with other heteroatoms, such as non-metallic atoms, to improve their surface chemistry [143], accelerate electron transfer, and enhance the catalytic reaction rate [144]. For instance, Zhu et al. prepared nitrogen-doped graphene (NG), nitrogen- and sulfur-co-doped graphene (NSG), and graphene oxide (GO) nanozymes by co-thermal oxidation of graphene oxide with urea and benzyl disulfide [96]. By doping nitrogen and sulfur atoms, they aimed to regulate the electronic structure and enhance the selectivity of the target, ultimately achieving synchronous and selective detection of five kinds of aromatic pesticides such as lactofen, etc. The detection range of various pesticides is 5–500 μM. When carbon-based nanozymes are doped with oxygen atoms, it is also beneficial to accelerate the electron transfer during the reaction. For instance, Dang et al. prepared phosphorus–oxygen-co-doped carbon nanosheets (POCNSs) by heating triphenylphosphine oxide (TPPO) and dipotassium hydrogen phosphate (K_2_HPO_4_) together in one step [97]. The P/O co-doping induced the asymmetric distribution of charge on the carbon skeleton, accelerated the adsorption and decomposition of H_2_O_2_, and at the same time, the nanosheet structure could expose more active sites, optimizing the catalytic pathway. This technique was applied for the detection of chlorpyrifos insecticides, and the LOD was as low as 0.31 μg/L. Additionally, Tao et al. synthesized Cl and O dual-doped nitrogen-doped porous carbon nanozymes (Cl_x_-pNC) by the NaCl melt-etching method [98], as shown in Figure 1A. The Cl/O dual doping could also promote the adsorption and decomposition of H_2_O_2_. Based on the alkaline phosphatase (ALP) activity inhibition–cascade catalytic amplification strategy, it achieved specific detection of glyphosate herbicide with a LOD as low as 0.07 μM.

In order to enhance the stability of nanozymes and enhance their catalytic activity in complex environments, carbon-based nanozymes can also be doped with other metal atoms. For instance, Wang et al. prepared manganese–nitrogen-co-doped carbon-based nanozymes (Mn@NC) by one-step pyrolysis using dimethylglyoxime (DICY), glucose, and manganese sulfate as precursors [99]. After freeze-drying treatment for 24 h to remove the water, they were pyrolyzed at 900 °C under a nitrogen stream for 2 h. The doping of Mn formed Mn-N active sites, promoting the catalytic decomposition of H_2_O_2_ and the efficiency of electron transfer. This team detected phoxim based on the molecular imprinting electrochemiluminescence (MIECL) mode. The MIECL detection limit was as low as 0.011 ng/mL, and the colorimetric detection limit was as low as 1.27 ng/mL. Zhang et al. enhanced the oxidase activity of the nanozyme by introducing Cu to form a Cu-N coordination structure [100] and endowed it with specific inhibitory effects on thiophanate-methyl (TM) insecticide. They prepared a copper-doped carbon-based nanozyme (Cu@NC) by a high-temperature pyrolysis method (Figure 1B). Using guanine and cuprous chloride (CuCl) as precursors, they mixed them and pyrolyzed them at 900 °C in a nitrogen stream for 2 h. Based on the specific inhibition of thiabendazole insecticide on the activity of Cu@NC, they constructed a colorimetric sensor, with a detection limit of 0.04 μg/mL, and realized on-site rapid detection using smartphone RGB analysis. It was proven that this sensor is suitable for the selective detection of TM in water and soil samples.

However, the non-uniform distribution of metal atoms in carbon-based nanozymes reduced their inhibition rate. So, single-atom nanozymes have been proposed to achieve high selectivity, reduced interference effect, and increased catalytic efficiency. For example, Liu et al. prepared cobalt single-atom nanozymes with an unsaturated coordination structure (SA-CoN_3_) through a two-step method [101]. First, Co/Zn dual-metal zeolite imidazolate frameworks (ZIFs) were synthesized, and then pyrolyzed at 900 °C in an argon atmosphere to form a three-coordinated cobalt single-atom structure, anchored in nitrogen-doped porous carbon. The unsaturated coordination increases the electronic density of cobalt atoms through electron-donating effects and accelerates the electron transfer rate, and the nitrogen accelerates oxygen adsorption and activation. Finally, the smartphone colorimetric platform is used to detect glyphosate herbicide. Using this technique, the LOD for glyphosate was 0.66 μM. Zhao et al. prepared Fe single-atom and Fe cluster Fe_AC_/Fe_SA_-NC nanozymes by loading Fe(II)–phenanthroline complexes onto the surface of ZIF-8 through high-temperature pyrolysis [102], as shown in Figure 1C. Based on the oxidase activity of Fe_AC_/Fe_SA_-NC and the inhibitory effect of thiols on Fe active sites, a fluorescence sensor can detect organophosphorus pesticides with a detection limit of 1.9 pg/mL. Wu et al. used a salt-template-assisted strategy and synthesized Cu-N-C single-atom nanozymes through the thermal decomposition of a mixture of CuCl_2_ and dicyandiamide [103], as shown in Figure 1D. Cu was first dispersed in ultrathin carbon nanosheets in the form of CuN_4_, then combined with natural enzymes (AChE and ChOx) to construct a cascade catalytic system for acetylcholine detection. Using this technique, organophosphate insecticides that inhibit AChE were detected at 0.60 ng/mL, with low matrix effects.

### 2.2. Metal-Based Nanozymes

Metal-based nanozymes are a class of nanomaterials with metal elements as their core, endowed with catalytic activity similar to enzymes through nano-structural design [145]. Due to their stable structures, good functionality, and ease of synthesis, they are the most commonly used nanozymes [146]. The structure of metal-based nanozymes can be optimized for the exposure degree of active sites and the efficiency of electron transfer by adjusting their size, morphology, and crystal planes, thereby enhancing catalytic performance [147]. The electronic density of active sites can also be regulated by introducing functional groups or heteroatoms [148]. Generally, metal-based nanozymes mainly fall into two types: single-metal-based nanozymes and multi-metal-based nanozymes.

#### 2.2.1. Single-Metal-Based Nanozyme

**Figure 2:**
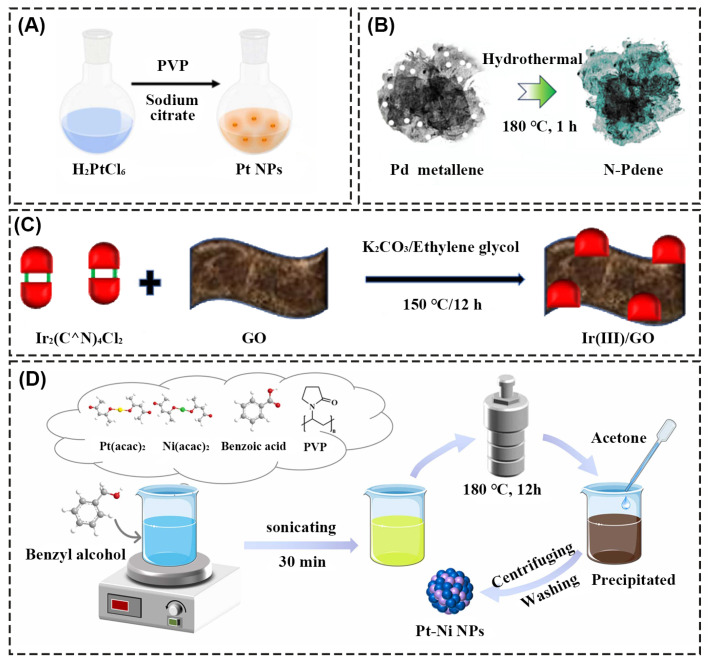
Schematic diagram of metal-based nanozyme synthesis. (**A**) Pt NPs. (**B**) N-Pdene. (**C**) Ir(III)/GO. (**D**) Pt-Ni NPs.

Single-metal-based nanozymes possess high catalytic activity, structural homogeneity, and stability [104]. Their clearly defined active sites and electronic density endow them with excellent selectivity [149]. Copper, having excellent biocompatibility and unique physicochemical properties, is widely used in the synthesis of single-metal nanozymes. For instance, Song et al. synthesized four copper-based nanozymes including guanosine 5′-monophosphate–copper (GMP-Cu), 4,4′-bipyridine–copper (BPY-Cu), 2-methylimidazole–copper (MIZ-Cu), and L-aspartic acid–copper (ASP-Cu) by coordinating copper ions with organic ligands (guanosine monophosphate, bipyridine, methylimidazole, aspartic acid) [104]. These nanozymes can reduce the cross-interference and matrix effects of the acetylcholinesterase method used for the determination of organophosphorus pesticide residues. Four-channel sensor arrays have been developed that use linear discriminant analysis (LDA) and hierarchical clustering analysis (HCA) to achieve selectivity for six organophosphorus pesticides. Platinum metal has also been used to construct nanozymes. For instance, Li et al. synthesized platinum nanoparticles (Pt NPs) with a particle size of 2.8 nm by using sodium citrate as the reducing agent and polyvinylpyrrolidone as the stabilizer through a chemical reduction method [105], as shown in Figure 2A. These Pt NPs exhibit broad-spectrum catalytic activity. A single reaction system can produce multiple channels of signals without the need for a multi-probe design. The sensitivity and catalytic activity of these nanozymes are 7 times higher than MnO_2_ nanosheets. This sensor array based on three-channel signals can distinguish five types of pesticides. The detection limit for dimethoate is 0.15 μg/mL in apple samples.

The enzymatic-like properties of nanomaterials can be enhanced by altering their lattice strain, porosity, and electronic structure. Aniqa et al. prepared Pd metalene nanozymes (N-Pdene) by a solvothermal method and atomic-scale reconstruction strategy [107], as shown in Figure 2B. To expand the lattice structure and enhance crystallinity, W(CO)_6_ and Pd(acac)_2_ were reacted in DMF and then N atoms from urea at high temperatures to form N-Pdene. N-Pdene exhibits catalytic activity similar to that of oxidase. Through lattice expansion and electronic structure modification, it activates O_2_ to generate reactive oxygen species (ROS) and catalyzes TMB coloration. A smartphone paper-based sensor was developed for glyphosate detection with a limit of detection of 0.27 μM, enabling low-cost and portable on-site detection. To reduce complexity in the synthesis of nanozymes, Wang et al. anchored the Ir(III) complex on the surface of GO through a coordination reaction between Ir and the oxygen-containing groups of GO [106]. This nanozyme (Ir(III)/GO) was synthesized in the absence of nitrogen doping or high-temperature pyrolysis, thus simplifying the synthesis steps (Figure 2C).

#### 2.2.2. Multi-Metal-Based Nanozyme

Multi-metal-based nanozymes enhance substrate adsorption and activation through the synergistic effects of multiple metals [150]. Alloy and core–shell structures increase the number of exposed surface atoms, providing more catalytic active sites and creating an enzyme-like microenvironment [151]. Multi-metal-based nanozymes thereby simultaneously mimic the functions of various natural enzymes, such as peroxidase, oxidase, and catalase [152]. Compared with single-metal-based nanozymes, multi-metal-based nanozymes distribute active sites through their core–shell and other structures, thereby reducing the usage of precious metals [153]. Additionally, the synergistic effects of different metal active sites can significantly enhance catalytic efficiency. For instance, Chen et al. synthesized Au@Pt core–shell nanozymes by a seed-mediated growth method [108]. With Au nanoparticles as the core, H_2_PtCl_6_ as the platinum source, and ascorbic acid (AA) as the reducing agent, a Pt layer was deposited on the surface of Au under gentle stirring to form a bimetallic structure. The catalytic mechanism of this nanozyme relied on the peroxide mimetic enzyme activity of Au@Pt, converting the non-fluorescent substrate Amplex Red (AR) into the fluorescent product thiazine. The catalytic activity was quantified by the change in fluorescence intensity. The advantages of this nanozyme include the synergistic enhancement of catalytic activity by the bimetallic system, the realization of multi-target detection by barcoding technology, and the improvement of detection specificity through magnetic separation technology. The detection limits for parathion, triazophos, and chlorpyrifos were 9.88 ng/kg, 3.91 ng/kg, and 1.47 ng/kg respectively, and the sensitivity was significantly superior to that of traditional immunoassay methods.

Similarly, by taking advantage of the peroxidase-like activity of multi-metal-based nanozymes, Zhao et al. synthesized PtPd bimetallic nanozymes by a co-reduction method [109]. Using Pluronic F127 as the surfactant template, K_2_PtCl_4_ and Na_2_PdCl_4_ as precursors, and AA as the reducing agent, the reaction was carried out in an ultrasonic water bath at 35 °C for 4 h. Finally, the particles were centrifuged for purification, and PtPd nanoparticles with a snowflake-like structure were obtained. Based on the peroxidase-like activity of PtPd nanozymes, they can efficiently catalyze the oxidation of catechol mediated by H_2_O_2_, thereby generating a dark-brown polymer and causing a change in the transmitted light intensity. With its high catalytic efficiency, the reusability of monoclonal antibodies, and the portability for smartphone detection, it is suitable for the rapid monitoring of organophosphorus pesticides.

Based on their oxidase-like activity, Jiang et al. synthesized platinum-nickel bimetallic nanoparticles (Pt-Ni NPs) by a one-step reduction method [110]. The precursors of platinum and nickel acetohydroxamic acid were mixed with PVP and benzoic acid in benzyl alcohol solvent. After a 12 h reaction at 180 °C, Pt-Ni NPs with an average particle size of approximately 6.15 nm were successfully prepared (Figure 2D). Pt-Ni NPs exhibited oxidase-like activity and could directly activate molecular oxygen to catalyze the oxidation of TMB to generate the blue product oxTMB without the participation of H_2_O_2_. Due to the photothermal effect of oxTMB under near-infrared light, dual-mode colorimetric/photothermal signal output can be achieved. The detection limits of the colorimetric and photothermal detection methods for chlorpyrifos were 1.2 ng/mL and 1.66 ng/mL, respectively.

### 2.3. Metal-Oxide-Based Nanozymes

Metal-oxide-based nanozymes are a kind of nanomaterial with natural enzyme-like activity. Their mechanism of action mainly relies on the electronic structure of surface functional groups, oxygen, and the variable oxidation state characteristics of metal ions [154]. For example, the oxygen on the surface of CeO_2_ can form a Ce^3+^/Ce^4+^ redox cycle, promoting the decomposition of H_2_O_2_ into H_2_O and O_2_, thereby simulating the activity of catalase [155]. These nanozymes have shown potential in cancer treatment, environmental remediation, and biosensing [156].

**Figure 3:**
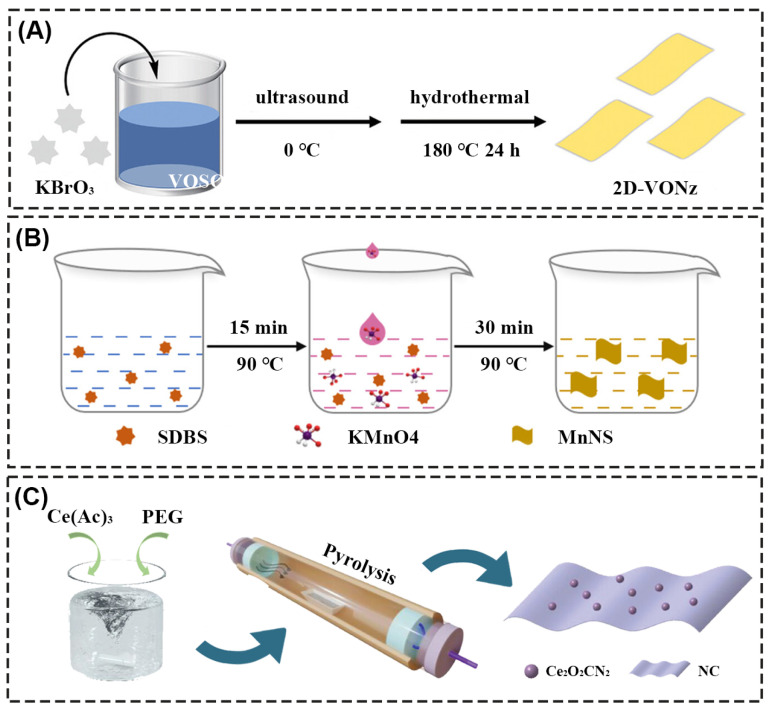
Schematic diagram of metal-oxide-based nanozyme synthesis; (**A**) 2D-VONz. (**B**) MnNS. (**C**) Ce_2_O_2_CN_2_/NC.

Metal-oxide-based nanozymes, through the synergy of multiple enzymatic activities, possess high stability, low toxicity, and multiple applications; these nanozymes show optimum results in food pesticide detection. For instance, Li et al. prepared two-dimensional V_2_O_5_ nanosheets (2D-VONz) by the reaction of VOSO_4_ and KBrO_3_ for the analysis of glyphosate herbicide [111], as shown in Figure 3A. These nanozymes generate ·OH radicals by decomposing H_2_O_2_, catalyzing the oxidation of the substrate TMB to trigger a color reaction. During the reaction, the π-π attraction between the substrate and the nanozyme surface enhances adsorption, leading to potential changes and promoting substrate contact with active sites, forming a dynamic adsorption–desorption equilibrium, which significantly improves catalytic efficiency. Due to its specific peroxidase activity, it can avoid O_2_ interference and specifically bind to glyphosate without the need for antibodies, enzymes, or aptamers, with a detection limit for glyphosate as low as 0.026 μM.

The two-dimensional structure can expose more active sites and have a greater catalytic rate and substrate affinity compared to traditional enzymes. Wu et al. also synthesized MnO_2_ nanosheets (MnNSs) with a two-dimensional structure by the sodium dodecyl benzene sulfonate (SDBS) template method [112], as shown in Figure 3B. They possess both oxidase and peroxidase activities, directly catalyzing substrate oxidation using O_2_, and further catalyzing substrate oxidation by utilizing the generated H_2_O_2_. This dual mechanism enables the synergistic effect of O_2_ and H_2_O_2_, significantly enhancing the catalytic efficiency. Based on electrochemical detection (DPV technique), this team replaced the colorimetric method and achieved a detection limit of paraoxon of 0.025 ng/mL, eliminating the interference from the color of the nanozyme itself and the color of the sample, and this method is suitable for the high-sensitivity detection of pesticide residues in complex samples. Compared with natural organophosphorus hydrolase (OPH) enzymes and CeO_2_ nanoparticles, Liu et al. synthesized Ce2O_2_CN_2_/NC nanozymes through a high-temperature pyrolysis strategy [113], for the detection of paraoxon insecticide; the details of the method are presented in Figure 3C. The affinity of this nanozyme is significantly higher than that of natural OPH, and the reaction rate is significantly higher than that of CeO_2_ nanoparticles. For the synthesis, urea is heated to 150 °C and then mixed with cerium acetate (Ce (OAc)_3_) and polyethylene glycol (PEG). The mixture is then pyrolyzed at 900 °C in an inert atmosphere. The nitrogen-doped carbon is used as a carrier, and the [N=C=N] ^2−^ groups decompose and are combined with cerium species to form uniformly dispersed Ce_2_O_2_CN_2_ nanoparticles. They have no response to interfering substances such as malathion and chlorpyrifos, and only specifically recognize the target containing phosphate ester bonds. The detection limit for paraoxon is 0.135 μM.

Nanoball-shaped nanozymes, due to their unique structure and properties, demonstrate significant advantages in catalytic efficiency, stability, and functional tunability. For instance, Jing et al. prepared Ag_2_O nanoballs by reacting AgNO_3_ with ammonia water [114]. Ag_2_O has oxidase activity and catalyzes the dissolution of O_2_ to generate O_2_^−^, thereby oxidizing TMB, o-phenylenediamine (OPD), and ABTS. This team constructed a sensor array through the colorimetric differences of TMB, OPD, and ABTS in three channels to achieve high selectivity for the differentiation of various organophosphorus pesticides. Combined with mobile phone RGB analysis for visual detection, it is applicable for on-site high-throughput detection and can simultaneously distinguish six organophosphorus pesticides, with a detection limit as low as 10 ng/mL.

### 2.4. MOF-Based Nanozymes

Metal–organic frameworks are a class of crystalline porous materials formed by the self-assembly of inorganic metal ions or metal clusters with organic ligands through coordination bonds [157]. The pore size and surface chemical properties can be precisely control by choosing appropriate metal nodes and ligands [158]. By introducing substances such as amines and metallization groups, specific functions such as catalysis and adsorption can be endowed to these nanozymes [159]. These materials have demonstrated efficient and stable catalytic performance in fields such as biological detection and cancer treatment [160]. The core mechanism lies in the synergy between atomic-level active sites and dynamic microenvironments [161].

**Figure 4:**
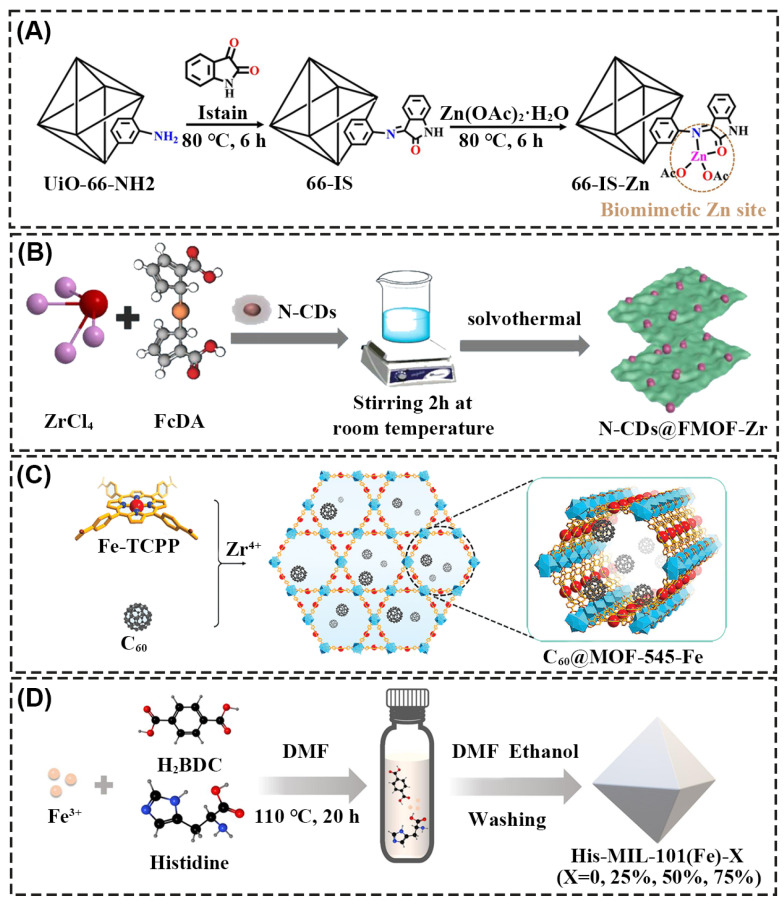
Schematic diagram of metal-oxide-based nanozyme synthesis: (**A**) 66-IS-Zn. (**B**) N-CDs/FMOF-Zr. (**C**) C_60_@MOF-545-Fe. (**D**) His-MIL-101(Fe)-X (X = 0, 25%, 50%, 75%).

MOF-based nanozymes generally achieve multi-enzyme activity and signal output through functionalization modification, such as metal site anchoring, host–guest encapsulation, and fluorescence molecule loading [162,163]. Due to these functionalities, these nanozymes are suitable for the high-sensitivity analysis of pesticide residues in complex matrices. For example, Huang et al. successfully synthesized 66-IS-Zn nanozymes with excellent catalytic performance by gradually modifying the biomimetic Zn sites anchored in metal–organic framework nanozymes [115], as shown in Figure 4A. Compared with the original UiO-66-NH2, the activity increased by 36 times and the Kcat value increased by 130 times, and it has both phosphatase and phosphodiesterase activities. Phosphatase-like activity can catalyze the hydrolysis of fluorescein diphosphate (FDP), releasing fluorescent products and generating detectable fluorescence signals. Phosphodiesterase-like activity can catalyze the hydrolysis of bis(4-nitrophenyl) phosphate (BNPP), generating p-nitrophenol that can cause changes in absorbance. Based on this principle, a multi-signal sensor was constructed for distinguishing six organophosphorus pesticides.

Unlike the complex process of stepwise modification, Luo et al. anchored nitrogen-doped carbon dots (N-CDs) in situ on Zr-based ferrocene metal–organic framework (FMOF-Zr) nanosheets through the solvothermal method to form N-CDs/FMOF-Zr nanozymes [164], as shown in Figure 4B. This simplifies the synthesis process of the nanozymes. Similarly, MOF-based nanozymes can simulate various enzymatic activities and significantly enhance catalytic efficiency. For instance, Bai et al. synthesized metal–organic framework C_60_@MOF-545-Fe nanozymes by a one-pot method [116], as shown in Figure 4C. The team utilized the strong π-π interaction between fullerene (C_60_) and the metal–organic framework MOF-545-Fe to encapsulate C_60_ in the pores of MOF-545-Fe, forming C_60_@MOF-545-Fe nanozymes. The uniform distribution of C_60_ within the MOF pores was verified by scanning electron microscopy, Raman spectroscopy, and X-ray diffraction. C_60_@MOF-545-Fe exhibits dual-enzymatic activities of oxidase and peroxidase. The dual-enzymatic activities can synergize and achieve cascade reactions without external energy input, demonstrating excellent detection performance for glyphosate, omethoate, and paraoxon, with detection limits of 0.65 ng/mL, 0.16 ng/mL, and 0.32 ng/mL, respectively. The team achieved multi-target discrimination through differential responses in a three-channel array and constructed a portable detection platform using deep learning algorithms, meeting the immediate detection requirements for trace pesticides in complex matrices.

The high stability and multifunctionality of MOF-based nanozymes are attracting more researchers to explore them. For instance, Liu et al. synthesized a bifunctional metal–organic framework nanozyme NH_2_-CuBDC by a solvothermal method [117]. The metal node was Cu^2+^, and the organic ligand was 2-aminoterephthalic acid (NH_2_-BDC). It was prepared by a solvothermal reaction assisted by PVP and maintained its activity in high-temperature and high-salt environments, outperforming natural HRP. This team utilized its oxidative enzyme-like activity to decompose H_2_O_2_ to generate superoxide radicals (·O_2_^−^), thereby oxidizing the chromogenic substrate TMB or fluorescent substrate OPD to achieve dual-mode detection. The detection limits for chlorpyrifos in colorimetric and fluorescence modes were 1.57 ng/mL and 2.33 ng/mL, respectively.

The enzyme-like activity of MOF nanozymes can be significantly enhanced by modifying the structural chemistry. For instance, Yue et al. modified MOF nanozymes with different amounts of histidine, namely His-MIL-101(Fe)-X (X = 0, 25%, 50%, 75%) [118], by using a one-step solvothermal method and optimizing the ligand ratio of histidine (His) and 2,5-dihydroxybenzoic acid (H_2_BDC), as shown in Figure 4D. The Lewis basicity of histidine regulates the electronic structure of Fe and reduces the substrate binding energy, thereby enhancing the catalytic efficiency. The doping of histidine increases the electronic density in the substrate through π-π interaction or hydrogen bonding, thereby accelerating the decomposition of H_2_O_2_ to generate ·OH radicals and enhancing the oxidation ability. Using the above principle, a three-channel (OPD/TMB/ABTS) sensor array was constructed by using His-MIL-101(Fe)-75%. The lowest detection limit of 2 μM was achieved for five pesticides, which was 25 times higher than that of unmodified nanozymes. The quantitative detection limit for thiourea pesticides such as diafenthiuron was 0.82 μM. Histidine modification significantly enhanced the peroxidase-like catalytic activity as compared to unmodified nanozymes and natural enzymes. By combining and evaluating the signal differences in the three substrates, the discrimination of multiple pesticides was easily achieved. The method demonstrated high sensitivity in soil and seawater matrices, and the operation was simple, providing an efficient solution for rapid on-site detection for pesticide residue monitoring.

### 2.5. Fluorescence-Based Nanozymes

Fluorescence-based nanozymes have become a research hotspot in the field of nanozymes in recent years due to their unique “self-luminescence” properties and multimodal detection capability [165]. The mechanism of fluorescence-based nanozymes mainly relies on the synergistic catalytic and fluorescence properties. Through the regulation of the electronic structure of the surface-active sites of the materials and the dynamic reaction pathways, they achieve multifunctional applications [166,167].

**Figure 5:**
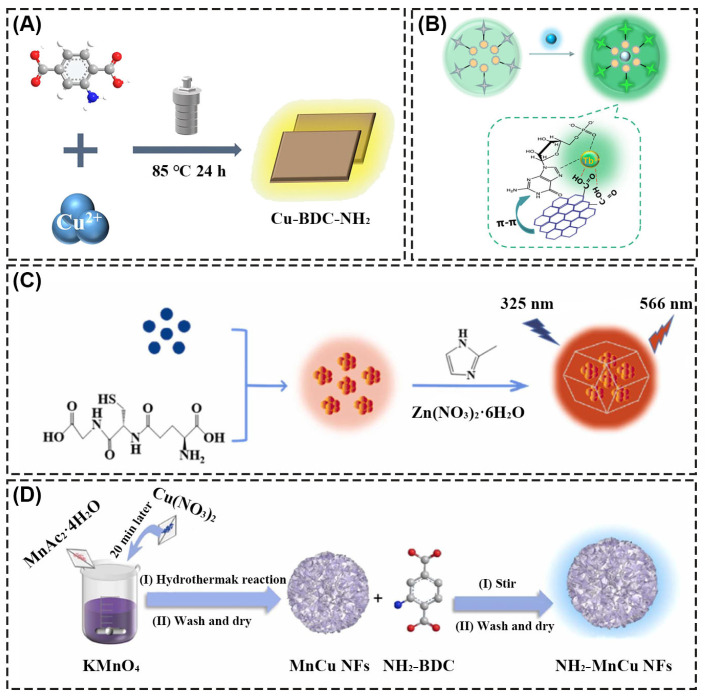
Schematic diagram of fluorescence-based nanozyme synthesis. (**A**) Cu-BDC-NH_2_. (**B**) GQD@Tb/GMP. (**C**) aZIF-8@CuNCs. (**D**) NH_2_-MnCu NFs.

Fluorescence-based nanozymes overcome the limitations of traditional pesticide detection methods, such as low sensitivity, complex operation, and high cost, through fluorescence signal amplification, anti-interference self-correction, and portable integrated design [168]. For instance, Song et al. synthesized the fluorescent nanozyme Cu-BDC-NH_2_ by a solvothermal method for the detection of 10 common and typical pesticides [119], as shown in Figure 5A. Using copper nitrate as the metal source, it was added to a mixture of NH_2_-BDC, DMF, and ethanol solvent, and reacted at 85 °C for 24 h. Finally, a crystal structure was formed through metal–organic coordination. Compared with natural enzymes, Cu-BDC-NH_2_ has a kcat of 0.119 mM and Vmax of 18.697 μM/min, which is superior to those of natural laccase, and has higher catalytic efficiency. Meanwhile, this nanozyme can maintain activity above 80% at high temperatures and extreme pH, demonstrating excellent stability. This team’s sensor array based on three signals achieved broad-spectrum identification of 10 types of pesticides. The linear discrimination analysis accuracy within the range of 1–100 μg/mL reached 100%. It can effectively identify multiple pesticide residues in real samples, with 100% accuracy and a minimum detection limit of 1 μg/mL. This provides a reliable solution for pesticide screening in complex pesticide mixtures.

The encapsulation of metal ions in the pores of the organic framework and on the surface also has an impact on the fluorescence intensity of the nanozymes. For example, Ma et al. synthesized Fe-doped Fe-CDs (carbon dots) by a hydrothermal method and combined them with the metal–organic framework MOF-808 to construct two types of fluorescent nanozymes [120], namely Fe-CDs encapsulated in MOF pores, with the Fe-CDs/MOF-808 nanozyme and Fe-CDs@MOF-808 nanozyme loaded on the surface. Fe-CDs/MOF-808 directly utilizes the enzyme-like activity of MOF-808 to hydrolyze paraoxon insecticide, while Fe-CDs@MOF-808 first converts the P=S bond into a P=O bond through photocatalytic oxidation. Using this technique, parathion insecticide was detected based on the two-step synergistic effect. The detection range of Fe-CDs/MOF-808 for paraoxon was 0.001–360 μM, and the detection limit was as low as 0.3 nM. The detection linear range of Fe-CDs@MOF-808 for parathion is 0.01–100 μM, and the detection limit is 3.3 nM, both of which are significantly similar to traditional chromatographic methods. This method was successfully applied to the rapid degradation, detection, and verification of the above-mentioned pesticide in real water and vegetable samples.

The encapsulation of GQDs can significantly increase the fluorescence intensity of nanozymes. For example, Ma et al. synthesized water-soluble GQDs by a hydrothermal method [123], with surface functional groups such as carboxyl and hydroxyl. Tb^3^⁺, guanosine monophosphate (GMP), and phosphate groups were reacted to form an infinite coordination polymer (ICP) nanoparticle. Finally, GQDs were encapsulated into Tb/GMP ICP through coordination between the carboxyl groups of GQDs and Tb^3^⁺. Non-covalent interactions (hydrogen bonds, π-π stacking) enhanced the host–guest binding, constructing the fluorescent GQD@Tb/GMP ICP nanozyme (Figure 5B). The coating of GQDs significantly enhanced the fluorescence of Tb^3^⁺, increased the quantum yield, and inhibited the aggregation-induced quenching (ACQ) effect of GQDs themselves. This team constructed a dual fluorescence ratio sensor based on GQD@Tb/GMP ICP, with a detection limit of 0.037 ng/mL for paraoxon, and combined it with smartphone technology to provide an innovative solution for immediate diagnosis and environmental monitoring.

By encapsulating metal clusters in amorphous MOFs, the fluorescence intensity of nanozymes can be significantly enhanced. For example, Yang et al. successfully synthesized aZIF-8@CuNCs fluorescent nanozymes by encapsulating stable glutathione-capped copper nanoclusters (CuNCs) in amorphous zeolite imidazole framework-8 (aZIF-8) through a one-pot method [121], as shown in Figure 5C. The fluorescence intensity was enhanced by 35 times through the confinement effect. This method utilized smartphone RGB analysis for visual detection, simplifying the on-site detection process. The detection results showed good linearity within the range of 0–50 μg/mL, with a detection limit of chlorpyrifos as low as 0.43 μg/mL. The method was successfully applied to the detection in food and soil samples. Yan et al. used KMnO_4_, MnAc_2_·4H_2_O, and Cu(NO_3_)_2_ as raw materials and conducted a hydrothermal reaction at 100 °C for 10 h to form manganese–copper nanoflowers (MnCu NFs) [122]. The negatively charged NH_2_-BDC was electrostatically adsorbed on the positively charged surface of MnCu NFs to form NH_2_-MnCu NF nanozymes (Figure 5D). NH_2_-MnCu NFs demonstrated high-sensitivity and dual-mode detection for organophosphorus pesticides and phenolic pollutants. With unique dual-enzymatic activities of OXD-like, laccase-like, and fluorescence functions, this nanozyme shows high selectivity, a low matrix effect, and practical application potential, providing an efficient multifunctional nanoplatform for environmental monitoring.

### 2.6. Other X-Based Nanozymes

**Figure 6:**
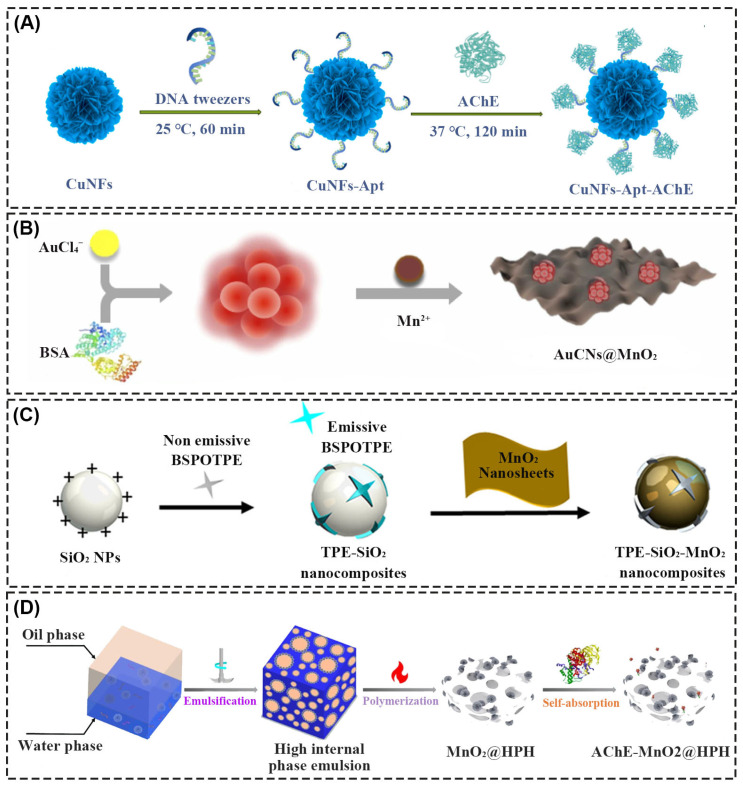
Schematic diagram of X-based nanozyme synthesis. (**A**) CuNFs-Apt-AChE. (**B**) AuNCs@MnO_2_. (**C**) BSPOTPE-SiO-MnO_2_. (**D**) AChE-MnO_2_@HPH.

Composite material-based nanozymes combine various multifunctional materials such as hydrogels, amino acids, and biomolecules in an integrated manner, demonstrating unique synergistic effects and multifunctionality [169,170,171]. They also have significant application advantages in the detection of pesticide residues in food. For instance, by integrating the signal amplification advantages of biomolecules with the stability of metal materials, Li et al. synthesized DNA-Cu nanoflowers (CuNFs) by a bio-mineralization method and constructed CuNFs-Apt-AChE composite materials based on DNA complementary pairing and aptamer technology [124], as shown in Figure 6A. In this method, G-DNA and copper ions were used to self-assemble in PBS to form flower-shaped CuNFs, and then aptamers (Apt) were connected through complementary sequences of DNA scissors to form CuNFs-Apt, and finally, acetylcholinesterase was specifically captured by aptamers to achieve precise binding of the enzyme and nanozyme. After embedding it into hydrogel, carbamate pesticide was detected using this sensor, with a detection limit of 0.19 ng/mL, which shows a high sensitivity and anti-interference ability.

By combining the high fluorescence stability of nanoclusters with the fluorescence quenching function of metal oxides, Li et al. adopted the co-template method to first synthesize gold nanoclusters (AuNCs) through the bovine serum albumin (BSA) bio-mimicry method [125]. Subsequently, Mn^2^⁺ was attached to BSA by coordination bonds and oxidized to generate MnO_2_ nanosheets under alkaline conditions. Finally, AuNCs@MnO_2_ composite material was formed (Figure 6B). A portable kit of AuNCs@MnO_2_ nanozymes in hydrogels was developed. Using this kit, paraoxon insecticide was detected at 5.0 ng/mL in Chinese cabbage. By integrating the high photostability of AIE molecules with the biocompatibility of metal oxides, Wu et al. successfully assembled BSPOTPE, SiO_2_ NPs, and MnO_2_ nanosheets through electrostatic adsorption to synthesize a sandwich nanocomposite material BSPOTPE-SiO-MnO_2_ [126], as shown in Figure 6C. MnO_2_ nanosheets were prepared by reacting H_2_SO_4_, SDS (dodecyl sulfate), and KMnO_4_ at 95 °C. SiO_2_ nanoparticles (NPs) were synthesized using tetraethyl orthosilicate (TEOS) and 3-aminopropyl triethoxysilane (APTES). The positively charged surface was used for the electrostatic adsorption of the negatively charged AIE molecule BSPOTPE. Based on this, a fluorescent on-site, rapid test strip was developed, which can detect paraoxon at 1 μg/L.

For instance, by integrating the biocompatibility of amino acids and the multivalent catalytic function of metal ions, Song et al. synthesized fibrous Asp-Cu nanozymes by using L-aspartic acid (Asp) and Cu^2+^ through coordination reactions under mild conditions [127]. These nanozymes exhibit laccase-like (LAC), peroxidase-like (POD), and superoxide dismutase-like (SOD) activities, which can catalyze the oxidation-coupling reactions of phenolic substrates to generate quinone-colored products, catalyze the decomposition of H_2_O_2_, oxidize TMB to generate blue oxTMB products, and inhibit NBT reduction to blue formazan based on Cu^2+^-mediated O_2_^−^ disproportionation to H_2_O_2_ and O_2_. This sensor array based on three enzyme activities was used for the broad-spectrum identification of eight pesticides, with a detection limit of 0.1 μg/mL. It also achieved 100% accurate identification of pesticides in unknown samples through a concentration-independent model, demonstrating low matrix effects and reliable detection performance in actual vegetable samples.

For instance, by integrating the stability of metal oxides with the multi-porous three-dimensional structure characteristics of hydrogels, Zhu et al. constructed a multi-level pore water gel composite nanozyme through a stepwise method [128], as shown in Figure 6D. Firstly, a MnO_2_ nanozyme with a flower-like microsphere structure was prepared by a hydrothermal reaction combined with calcination using oleic acid as the reducing agent and morphology regulator. Then, this microsphere was used as the emulsion stabilizer to prepare the MnO_2_@HPH porous water gel scaffold by the high-internal-phase emulsion template thermal polymerization method. Finally, acetylcholinesterase was immobilized in the water gel pores by the rapid self-absorption method to form the AChE-MnO_2_@HPH composite nanozyme. Based on its oxidative enzyme mimetic activity, this team activated dissolved oxygen to generate superoxide free radicals to catalyze the color development reaction of TMB, achieving excellent detection performance for fenthion. The detection limit was as low as 0.63 ng/mL, and the linear range covered 4–400 ng/mL. It was successfully applied for the detection of fenthion in actual grain samples.

For instance, by integrating the magnetic separation characteristics of metal oxides and the catalytic enhancement function of metals, Deng et al. successfully synthesized Fe_3_O_4_@Cu composite nanozymes by combining metal oxides and metals, using solvothermal and chemical reduction methods [129]. Firstly, Fe_3_O_4_ nanoparticles were prepared by the solvothermal method at 200 °C for 9 h. Then, copper nanoparticles were loaded onto the surface of Fe_3_O_4_ in the presence of NaBH_4_ and CuSO_4_. This nanozyme, based on its peroxidase-like activity, can catalyze the decomposition of H_2_O_2_ to produce hydroxyl radicals ·OH, and then oxidize the chromogenic substrate TMB to generate blue oxTMB or excite luminol to produce chemiluminescence. The synergistic effect of Cu and Fe_3_O_4_ increased the specific activity by 2 times compared with Fe_3_O_4_ alone, and its affinity for H_2_O_2_ and TMB was significantly better than most similar nanozymes. Due to its dual mode, the detection limit was 0.086 μg/mL and 0.019 μg/mL for colorimetric and chemiluminescence methods, respectively. This method was successfully applied to the detection of glyphosate in actual samples, with an average recovery of 91.8–109.6%, showing its potential application for the on-site detection of pesticides in agricultural products.

## 3. Multimodal Sensing Technology

At present, single-modal sensing technology based on nanozymes has been widely studied [172,173,174]. Compared with a single signal, which is easily affected by external factors, multimodal sensing technology combines the catalytic-like activity of nanozymes with multiple signal detection modes [175,176,177]. By using different signals to mutually verify and improve the accuracy of the results, it can significantly enhance the sensitivity, selectivity, and specificity of detection [178,179], thus effectively avoiding the influence of external factors and having broader application prospects in complex real-world detection environments [180]. In this section, we introduce current multimodal sensing technologies based on nanozymes, mainly including colorimetry/fluorescence sensing, fluorescence/photothermal sensing, photothermal/colorimetry sensing, and other multimodal sensing techniques. Table 2 lists the multimodal sensing techniques used for pesticide detection.

### 3.1. Colorimetric/Fluorescence Sensing

Colorimetric sensing and fluorescence sensing are currently the two most widely used sensing methods in detection [181,182,183,184]. Colorimetric sensing is based on the formation of colored compounds through a color-developing reaction. Quantitative analysis is achieved by comparing the color changes of the solution or measuring the absorbance [185,186]. In fluorescence sensing, the analyte of interest absorbs specific wavelengths of light to excite electrons to the excited state; subsequently, the analyte releases energy in the form of fluorescence when the electrons come to the ground state. High-sensitivity detection is achieved by measuring the correlation between fluorescence intensity and analyte concentration [187,188].

**Figure 7:**
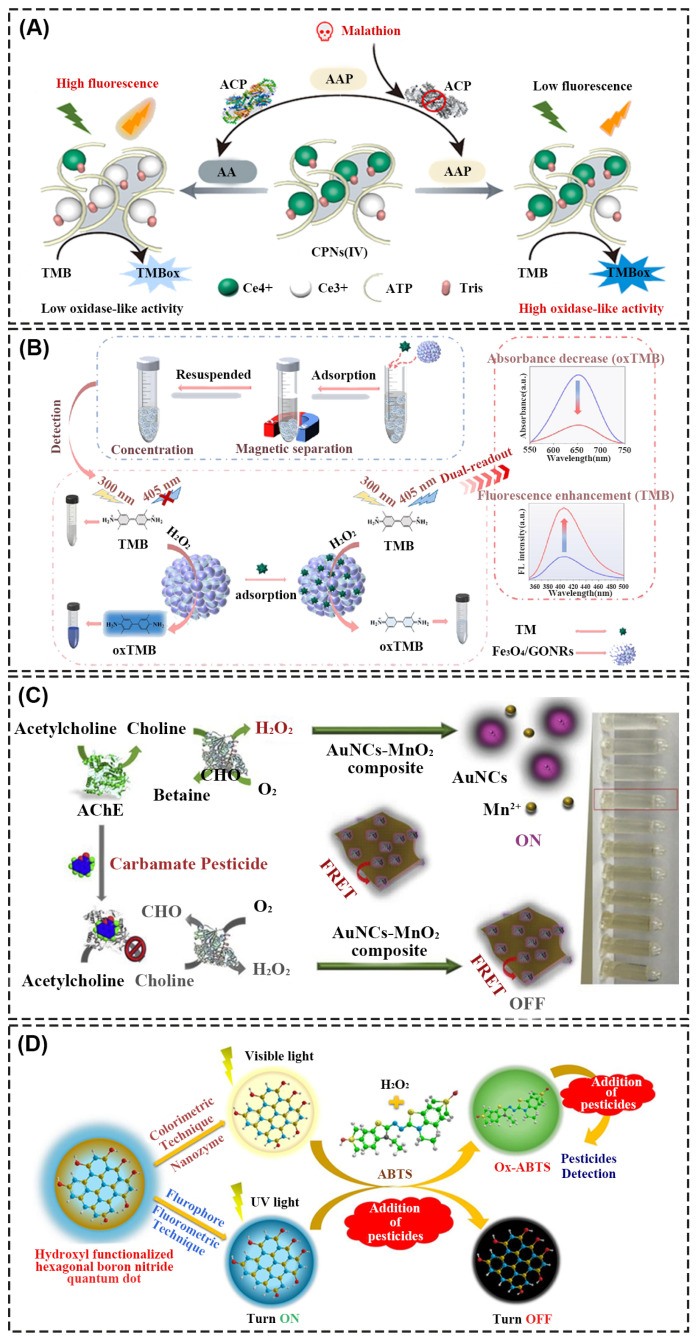
Schematic diagram of colorimetric/fluorescence dual-mode sensing. (**A**) Colorimetric and fluorescence dual-mode sensing based on CPNs(IV). (**B**) Colorimetric and fluorescence dual-mode sensing based on Fe_3_O_4_/GONRs. (**C**) Colorimetric and fluorescence dual-mode sensing based on AuNCs-MnO_2_. (**D**) Colorimetric and fluorescence dual-mode sensing based on h-BN QDs.

Colorimetric sensing and fluorescence sensing are currently the two most widely applied sensing methods for the detection of pesticide residues [189]. By combining the results of these two techniques, we can significantly improve the detection reliability by cross-validation and reduce the rate of false-positive and -negative results. For instance, Liu et al. proposed a dual-mode pesticide detection method based on cerium-based coordination polymer nanoparticles (CPNs(IV)) that combines fluorescence and colorimetry [190], as shown in Figure 7A. The colorimetric detection is based on the peroxidase-like activity of CPNs(IV), which can catalyze the oxidation of the colorless TMB to blue oxTMB in the presence of dissolved oxygen, generating a characteristic absorption peak at 652 nm. When acid phosphatase (ACP) and ascorbic acid-2-phosphate (AAP) are added, ACP hydrolyzes AAP to generate AA, which reduces Ce^4+^ in CPNs(IV) to Ce^3+^, forming CPNs(III) with enhanced fluorescence but lost peroxidase activity, thereby inhibiting the colorimetric signal. The fluorescence detection relies on the strong fluorescence of CPNs(III) at 356 nm. When malathion is present, it inhibits ACP activity, blocks the conversion of AAP to AA, and thus prevents CPNs(IV) from being reduced to CPNs(III), so the colorimetric signal is restored while the fluorescence signal is suppressed, achieving ‘off–on–off’ fluorescence and ‘on–off–on’ colorimetric dual-mode detection. The colorimetric and fluorescence signals are independent of each other. Through cross-validation, the detection reliability is improved. Ultimately, the detection limit of malathion by the colorimetric method is 0.032 μg/mL, and that of the fluorescence method is 0.046 μg/mL, which is superior to most single-mode sensors. Moreover, this method can be extended to the detection of other organophosphorus or carbamate pesticides that inhibit ACP activity, demonstrating good universality.

**Table 2:** Multimodal sensing techniques used for pesticide detection.

Multimodal Sensors	Nanozyme	Detected Pesticides	Sample	Sensors	Linear Range	Detection Limit	Ref.
Colorimetric/Fluorescence	CPNs(Ⅳ)	Malathion	Unhulled Rice Cucumber	Colorimetric	0.06–3 μg mL^−1^	0.032 μg mL^−1^	[190]
				Fluorescence	0.062–15.46 μg mL^−1^	0.046 μg mL^−1^	
	Fe_3_O_4_/GONRs	Thiophanate-methyl	Apple Water Rice Cabbages	Colorimetric	0.05–8 μg mL^−1^	28.1 ng mL^−1^	[191]
				Fluorescence	0.02–10 μg mL^−1^	8.81 ng mL^−1^	
	AuNCs-MnO_2_	Carbaryl	Water, Soil Rice, Apple	Colorimetric	/	12.5 μg mL^−1^	[192]
				Fluorescence	0.125–750 μg L^−1^	0.125 μg L^−1^	
	h-BN QDs	Atrazine Simazine Chlorpyrifos	Cucumbers Tomatoes Grapes Mangoes	Colorimetric	0–400 µM	2.47 µM	[193]
				Fluorescence	0–600 µM	8.08 µM	
Fluorescence/Photothermal	PDA NPs	Dimethoate	Water	Fluorescence	1–100 μM	0.1 μM	[194]
				Photothermal	1–2000 μM	0.1 μM	
	MnO_2_NSs	Dichlorvos Chlorpyrifos	Tea Brown Rice	Fluorescence	0.1–8000 ng mL^−1^	0.86 ng mL^−1^	[195]
				Photothermal	10–10,000 ng mL^−1^	1.01 ng mL^−1^	
	MnO_2_NSs	Isocarbophos	Apple Cowpea Water	Fluorescence	0.24–7.81 ng mL^−1^	0.21 pg mL^−1^	[196]
				Photothermal	41.15–10,000 ng mL^−1^	2.62 pg mL^−1^	
	ZIF-Co-Cys	Dimethyl Dichloroviny Phosphate	Tea Brown Rice Wheat Flour	Fluorescence	2–100 ng mL^−1^	1.64 ng mL^−1^	[197]
				Photothermal	10–10,000 ng mL^−1^	0.084 ng mL^−1^	
Photothermal/Colorimetric	PCN-224-Mn	Glyphosate	Tea Brown Rice Wheat Flour	Photothermal	5–10,000 ng mL^−1^	2.00 ng mL^−1^	[198]
				Colorimetric	5–10,000 ng mL^−1^	1.47 ng mL^−1^	
	2D-ZHM	Glyphosate Omethoate	Water Apple Pear	Photothermal	0.04–2 nM	0.02 nM	[199]
				Colorimetric	0.01–1 nM	0.004 nM	
	ZrFc@CPN	Glyphosate	Rice Millet Soybean	Photothermal	0.039–20.16 μg mL^−1^	0.0289 μg mL^−1^	[200]
				Colorimetric	0.039–20.49 μg mL^−1^	0.0326 μg mL^−1^	
Other multimodal sensors	ACC-HNFs	Paraoxon	Water Rice Apple	Colorimetric	0.01–100 ng mL^−1^	10 pg mL^−1^	[201]
				Electrochemical	6.0 × 10^−6^–0.6 ng mL^−1^	6 fg mL^−1^	
	COF/MB@MnO_2_	Chlorpyrifos	Water Cabbages Chinese Chives Apples	Photothermal	0–8000 ng mL^−1^	0.108 ng mL^−1^	[202]
				Electrochemical	0.5–200 ng mL^−1^	0.0632 ng mL^−1^	
	MO@FHO	Malathion	Spinach	Colorimetric	0.001–50 μmol/L	0.26 ng mL^−1^	[203]
				Photoelectrochemical	0.0001–0.5 μmol/L	0.017 ng mL^−1^	
	N-CDs/FMOF-Zr	Glyphosate	Rice Millet Soybean	Colorimetric	0.039–3.19 µg mL^−1^	0.0131 µg mL^−1^	[164]
				Fluorescence	0.0088–3.98 µg mL^−1^	0.0015 µg mL^−1^	
				Photothermal	0.016–3.98 µg mL^−1^	0.0115 µg mL^−1^	

Considering that TMB emits fluorescence at 405 nm under 300 nm excitation, Tai et al. synthesized a spherical Fe_3_O_4_/GONRs composite nanozyme and constructed a colorimetric and fluorescence dual-mode detection method for TM [191], as shown in Figure 7B. This nanozyme has good catalytic activities of peroxidase and catalase, and it can catalyze H_2_O_2_ to oxidize the colorless TMB into blue oxTMB. Since TM is adsorbed on the surface of Fe_3_O_4_/GONRs by the synergistic action of various forces, the catalytic activity of the nanozyme is inhibited. This inhibition will cause a dual response of decreased absorbance and enhanced fluorescence, thereby achieving dual-mode detection. The detection limits of thiophanate methyl in colorimetric and fluorescence methods are 28.1 ng/mL and 8.81 ng/mL, respectively.

Furthermore, the dual-mode system can adapt to complex sample matrices, and it integrates colorimetric and fluorescence sensing, which complement each other to broaden the detection range [204]. It is suitable for high-accuracy analysis requirements in various scenarios. For instance, Yan et al. designed a multi-signal sensitive detection platform for carbamate pesticides based on AuNCs-MnO_2_ nanocomposite materials [192], as shown in Figure 7C. The colorimetric detection is based on the characteristic absorption peak of MnO_2_ nanosheets at 350 nm, and its decomposition leads to a decrease in absorbance and a change in the solution color from brownish to colorless. When carbamate pesticides are present, MnO_2_ decomposition is inhibited, and the absorbance remains at a high level. The fluorescence detection is based on the fluorescence of AuNCs, which can be quenched by MnO_2_ nanosheets through the Förster resonance energy transfer (FRET) effect. When AChE/ChOx double enzymes catalyze the substrate ACh to generate H_2_O_2_, H_2_O_2_ decomposes MnO_2_ into Mn^2+^, disrupting the FRET effect and restoring the fluorescence of AuNCs. Carbamate pesticides can inhibit AChE activity, reducing H_2_O_2_ generation and thereby inhibiting fluorescence recovery. The degree of color change of this sensor is positively correlated with the pesticide concentration, while the fluorescence intensity is negatively correlated with the concentration. The detection limits of the colorimetric and fluorescence methods are 12.5 μg/L and 0.125 μg/L, respectively, providing a reliable solution for the on-site rapid detection of carbaryl residues in agricultural products.

In the presence of organophosphorus and organochlorine pesticides in aqueous medium, Chayanika et al. developed a colorimetric and fluorescence dual-mode sensor based on hydroxyl-functionalized hexagonal boron nitride quantum dots (h-BN QDs) [193], as shown in Figure 7D. The colorimetric detection was based on the peroxidase-like activity of h-BN QDs, which catalyzed H_2_O_2_ oxidation of ABTS to generate the green product oxABTS under acidic conditions. The fluorescence detection was based on the emission of 393 nm fluorescence from h-BN QDs at 280 nm excitation. When pesticides were present, they inhibited the oxidation of ABTS through hydrogen bonds and π-π interactions, resulting in a decrease in absorbance. Meanwhile, the pesticide molecules caused fluorescence quenching through non-covalent interactions of surface hydroxyl groups and boron/nitrogen atoms, ultimately achieving dual-mode detection. The detection limits of the sensor for atrazine, simazine, and chlorpyrifos were 6.11, 6.56, and 2.47 µM by colorimetry, and 5.59, 6.45, and 8.08 µM by fluorescence. The sensor showed no obvious response to interfering ions such as Ag^+^ and Cd^2+^ and had strong specificity. Moreover, h-BN QDs had excellent water dispersibility, and the fluorescence intensity did not significantly decay within 90 days. It also had no heavy metal toxicity and was suitable for environmental monitoring and food safety fields.

### 3.2. Fluorescence/Photothermal Sensing

The pesticide photothermal detection method based on nanozymes achieves sensitive detection of target analytes by taking advantage of the catalytic activity of nanomaterials similar to enzymes and their photothermal conversion characteristics [205]. For instance, nanozymes catalyze the generation of TMB, which produces an oxidized product oxTMB with near-infrared absorption ability [206]. Under near-infrared light irradiation, this method can efficiently convert light energy into thermal energy, causing a significant increase in solution temperature and generating a quantifiable photothermal signal [207,208].

Currently, the dual-mode sensing detection of pesticide residues by fluorescence and photothermal has become a research hotspot [209,210]. Based on the catalytic characteristics of mimetic enzymes and the dual-mode signal amplification effect of photothermal and fluorescence detection, quantitative analysis of pesticides can be carried out while significantly improving the sensitivity. For example, Liu et al. established a fluorescence and photothermal dual-mode analysis method for detecting organophosphorus pesticides by inducing the formation of polydopamine (PDA) nanoparticles with ALP inhibitors [194]. This team utilized MnO_2_ nanosheets to oxidize dopamine (DA) and form PDA nanoparticles with fluorescence and excellent photothermal conversion efficiency. Based on the principle that AA is produced by ALP catalyzing AAP, AA reduces MnO_2_ to Mn^2+^, inhibiting DA oxidation and reducing PDA formation, thereby achieving fluorescence detection. Photothermal detection is achieved by generating PDA with a significant temperature increase under near-infrared light (780 nm) irradiation. When dimethoate, an organophosphate insecticide, is present, AA is inhibited from being generated, resulting in a significant increase in fluorescence intensity and solution temperature after a large amount of PDA is generated. Both modes have a detection limit of 0.1 µM for dimethoate and have been successfully used for the detection of river water and tap water samples. They show no obvious reactions to triazoles, organochlorines, and common ions (Na⁺, K⁺, etc.), and only respond to organophosphorus pesticides, demonstrating good sensitivity for this class.

**Figure 8:**
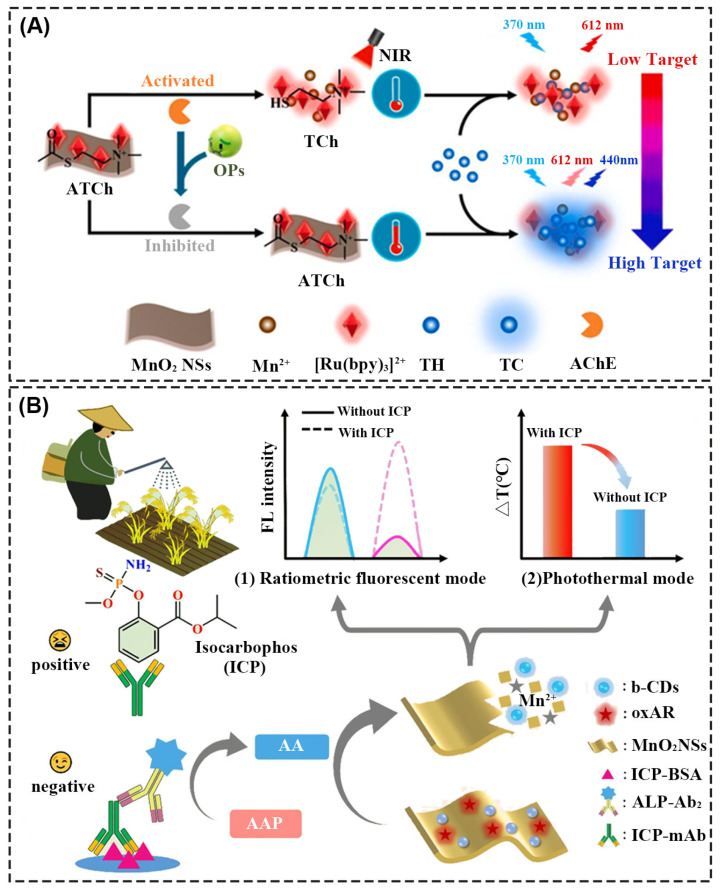
Schematic diagram of fluorescence/photothermal dual-mode sensing. (**A**) Fluorescence and photothermal dual-mode sensing based on MnO_2_NSs. (**B**) Fluorescence and photothermal dual-mode sensing based on b-CDs.

Similarly, by utilizing Mn^2+^ in catalytic reactions, Jiang et al. constructed a ratio fluorescence and photothermal dual-mode probe based on manganese oxide nanosheets (MnO_2_NSs) for the quantitative detection of organophosphorus pesticide residues [195], as shown in Figure 8A. When there was no pesticide, AChE catalyzed the hydrolysis of ATP to generate TCh, which reduced MnO_2_NSs to Mn^2+^, and the residual Mn^2+^ had no oxidase activity and could not oxidize thiamine. At this time, the red fluorescence of the reference signal [Ru(bpy)_3_]^2+^ dominated. When there was pesticide, the pesticide inhibited AChE activity, resulting in a decrease in TCh production and an increase in the residual MnO_2_NSs.

MnO_2_NSs oxidized the non-fluorescent thiamine to the blue fluorescent product thiochrome through enzymatic activity and partially quenched the red fluorescence of [Ru(bpy)_3_]^2+^ through the FRET effect. Finally, the fluorescence intensity ratio was used to achieve dichlorvos and chlorpyrifos concentration detection. The photothermal detection was based on the efficient photothermal conversion performance of MnO_2_NSs under 808 nm near-infrared laser irradiation. When there was no pesticide, MnO_2_NSs were reduced to Mn^2+^ by TCh, and the solution temperature increase was small. Conversely, the residual MnO_2_NSs increased, and the photothermal effect enhanced, resulting in a significant increase in solution temperature. Infrared thermography was used to record the temperature changes of the solution to achieve pesticide concentration detection. This probe exhibited good anti-interference and reliability in real samples. The dual-mode probe with self-calibration functionality is expected to provide more accurate and robust detection results than single-mode probes and has broader application prospects.

Based on the photothermal effect of MnO_2_NSs itself, Liu et al. utilized its ability to oxidize the non-fluorescent Amplex Red (AR) to generate the red-fluorescent oxidized product oxAR [196], and the principle of the internal filter effect (IFE) caused the quenching of the fluorescence of blue carbon dots (b-CDs). They constructed a dual-mode immunosensor, as shown in Figure 8B. Using ALP as the marker enzyme to catalyze AAP to produce AA, the generated AA can decompose MnO_2_NSs, resulting in a reduction in the photothermal signal of the nanozyme and the fluorescence signal of oxAR, while moderately restoring the fluorescence emission of b-CDs. When isocarbophos pesticides are present, AA is inhibited from being generated, and the above signals are reversed and enhanced, ultimately achieving dual-mode detection. The detection limit in photothermal mode is as low as 2.62 pg/mL, and in ratio fluorescence mode, it is 0.21 pg/mL. Acceptable recoveries were obtained in real samples of apples, cowpeas, and tap water, demonstrating the applicability of this method.

Similarly, by taking advantage of the inhibitory effect of pesticides on the enzymatic activity of ACP, Zhang et al. prepared ZIF-Co-Cys nanozymes with high catalytic activity of peroxidase-like enzymes [197], and based on the competitive effect of the products of the enzymatic reaction of ZIF-Co-Cys with ACP and o-phenylenediamine (OPD), they constructed a ratio fluorescence and photothermal dual-mode probe for the detection of organophosphorus pesticides. The fluorescence detection was based on the hydrolysis of AAP by ACP to generate AA, which reacted with OPD to form the fluorescent product 3-(1,2-dihydroxyethyl) furan [3,4-b] quinoline-1(3H) (DFQ), while the ZIF-Co-Cys nanozyme could catalyze the generation of the fluorescent product 2,3-diaminophenazine (DAP) from OPD, which could compete with DFQ to consume OPD. When pesticides were present, the AA generation was inhibited, and DFQ decreased, while DAP increased. The detection of dichlorvos insecticide was achieved based on the fluorescence intensity ratio. The detection limit was 1.64 ng/mL. Due to the self-aggregation of DAP under the catalysis of ZIF-Co-Cys to form OPD polymer nanoparticles, when irradiated with an 808 nm laser, the nanoparticles converted the light energy to heat energy through non-radiative transitions, resulting in a significant increase in solution temperature and enabling photothermal detection. The detection limit was 0.084 ng/mL in the photothermal method, providing a new idea for the design of multimodal sensing platforms based on nanozyme cascade reactions.

### 3.3. Photothermal/Colorimetric Sensing

**Figure 9:**
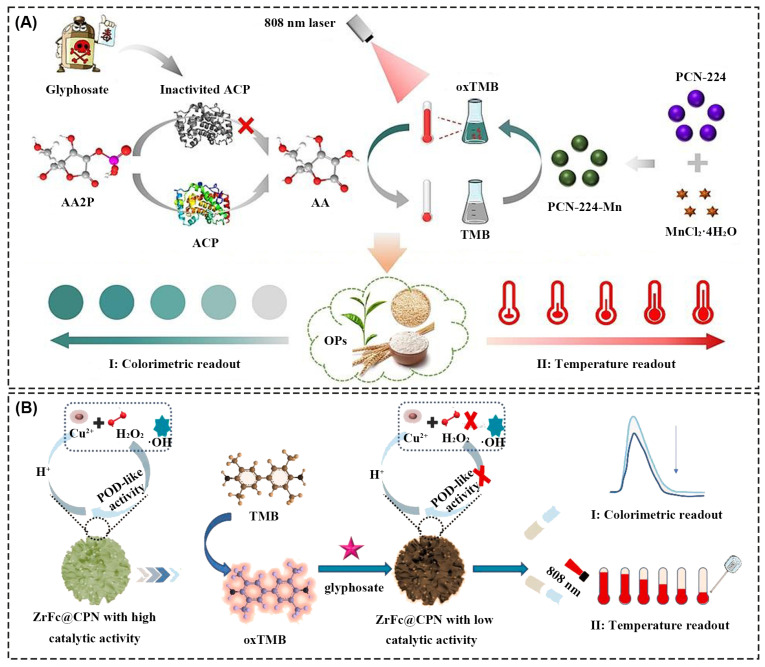
Schematic diagram of photothermal/colorimetric dual-mode sensing. (**A**) Photothermal and colorimetric dual-mode sensing based on PCN-224-Mn. (**B**) Photothermal and colorimetric dual-mode sensing based on ZrFc@CPN.

Photothermal sensing can achieve quantitative analysis with anti-background interference by utilizing the photothermal effect of oxidized substrates, and colorimetric sensing can provide intuitive visual results, so multimodal sensing based on photothermal colorimetry has been extensively studied [211,212]. Cross-signal verification significantly improves the detection reliability and enables the trace detection of pesticides in complex matrices, providing a new direction for on-site rapid detection [213]. For example, Wang et al. developed a new dual-mode probe for the colorimetric and photothermal detection of organophosphorus pesticides based on a manganese-modified porphyrin metal–organic framework (PCN-224-Mn) [198], as shown in Figure 9A. Colorimetric sensing is based on the catalytic activity of PCN-224-Mn similar to that of oxidase, which can catalyze the oxidation of colorless TMB to blue oxTMB. When there is no pesticide, ACP catalyzes the generation of AA from AAP, inhibiting the oxidation of TMB, resulting in a decrease in absorbance. When there is pesticide, the generation of AA is inhibited, and the TMB oxidation reaction is restored, resulting in an increase in absorbance. Photothermal sensing is based on the photothermal conversion ability of oxTMB under 808 nm near-infrared light irradiation, causing the solution temperature to rise, and the final temperature change is positively correlated with the pesticide concentration. This dual-mode colorimetric and photothermal detection platform has a detection limit of 1.47 ng/mL and 2.00 ng/mL for glyphosate herbicide. This method has been successfully used for the detection of organophosphorus pesticide residues in tea, brown rice, and wheat flour, and the detection results can be read through a smartphone, making it suitable for on-site rapid detection.

Using the same principle of direct catalysis of TMB, Shen et al. used H_2_O_2_ to indirectly oxidize TMB [199]. Based on the prepared 2D hemin-bridged MOF nanozyme (2D-ZHM), they developed a colorimetric photothermal dual-mode sensing platform for organophosphorus pesticides. The colorimetric sensing is based on the peroxidase-like activity of 2D-ZHM, which catalyzes the oxidation of H_2_O_2_ to generate oxTMB. When there is no organophosphorus pesticide, the organic ligand heme binds to Zr^4+^ through the phosphate backbone and covers the catalytic active site, inhibiting the oxidation of TMB and reducing the absorbance. When there is an organophosphorus pesticide, heme binds to OPs and detaches from the surface of 2D-ZHM, and the nanozyme regains catalytic activity, restoring the oxidation of TMB, and the absorbance increases with an increase in OP concentration. This colorimetric and photothermal dual-mode sensing platform has a detection limit of 0.004 nM for glyphosate and 0.02 nM for omethoate, demonstrating significant accuracy, sensitivity, specificity, and robustness, and expanding the rational design of high-performance and MOF nanozymes.

Similarly, Luo et al. constructed a colorimetric and photothermal dual-mode detection method for glyphosate based on ZrFc@CPN nanozyme [200], as shown in Figure 9B, and solved the problems of single-signal responses and the need for additional H_2_O_2_. This approach improved detection stability while simplifying the operation process and reducing toxicity risks. Due to the copper peroxide nanodots (CPNs) being able to trigger Fenton-like catalysis under acidic conditions (pH = 4.0), they release H_2_O_2_ and Cu^2+^, and the peroxidase activity of ZrFc-MOF further catalyzes the decomposition of H_2_O_2_, achieving a cascade effect. Eventually, TMB can be oxidized to oxTMB, generating colorimetric and photothermal temperature signals, enabling dual-mode sensing. The detection limits for glyphosate were 0.0326 µg/mL and 0.0289 µg/mL, respectively. The ZrFc@CPN nanozyme used in this method provides self-supply of H_2_O_2_ and cascade catalysis through its hierarchical structure and dual-active-site design. Its recovery rate reached 90.94–110.57% in rice, millet, and soybeans, and it has broad application prospects in food and environmental monitoring.

### 3.4. Other Multimodal Sensing

Apart from the aforementioned colorimetric sensing, fluorescence sensing, and photothermal sensing, many researchers have developed various sensing technologies based on these approaches, such as electrochemical sensing [214,215,216], photoelectrochemical sensing [217], etc. For instance, Jin et al. constructed a dual-mode paper-based biosensor for colorimetric and electrochemical sensing based on all-in-one enzyme–inorganic hybrid nanoflowers (ACC-HNFs) [201]. The colorimetric sensing is based on the peroxidase-like catalytic activity of ACC-HNFs, which catalyzes the oxidation of H_2_O_2_ by TMB to produce a blue color. The electrochemical sensing is based on the fact that the imine product of TMB oxidation turns yellow and becomes positively charged under acidic conditions, and it can undergo redox reactions on the electrode surface. When the test paper is folded and comes into contact with the screen-printed carbon electrode (SPCE), the imine directly transfers electrons to the electrode, and the current change is measured by amperometry. This paper-based sensor has a detection limit of 10 pg/mL for colorimetric sensing and 6 fg/mL for electrochemical sensing of peroxymonosulfate (POMS). Due to the sensitivity of our eyes being limited to discerning obvious color changes, the semi-quantitative detection of POMS can reach 0.5 µg/mL. This paper-based biosensor eliminates the need for complex sample pretreatment and precise instruments for detecting signals, and is suitable for on-site monitoring. Its ultra-sensitivity and high selectivity provide innovative solutions for the analysis of trace pollutants in complex matrices.

**Figure 10:**
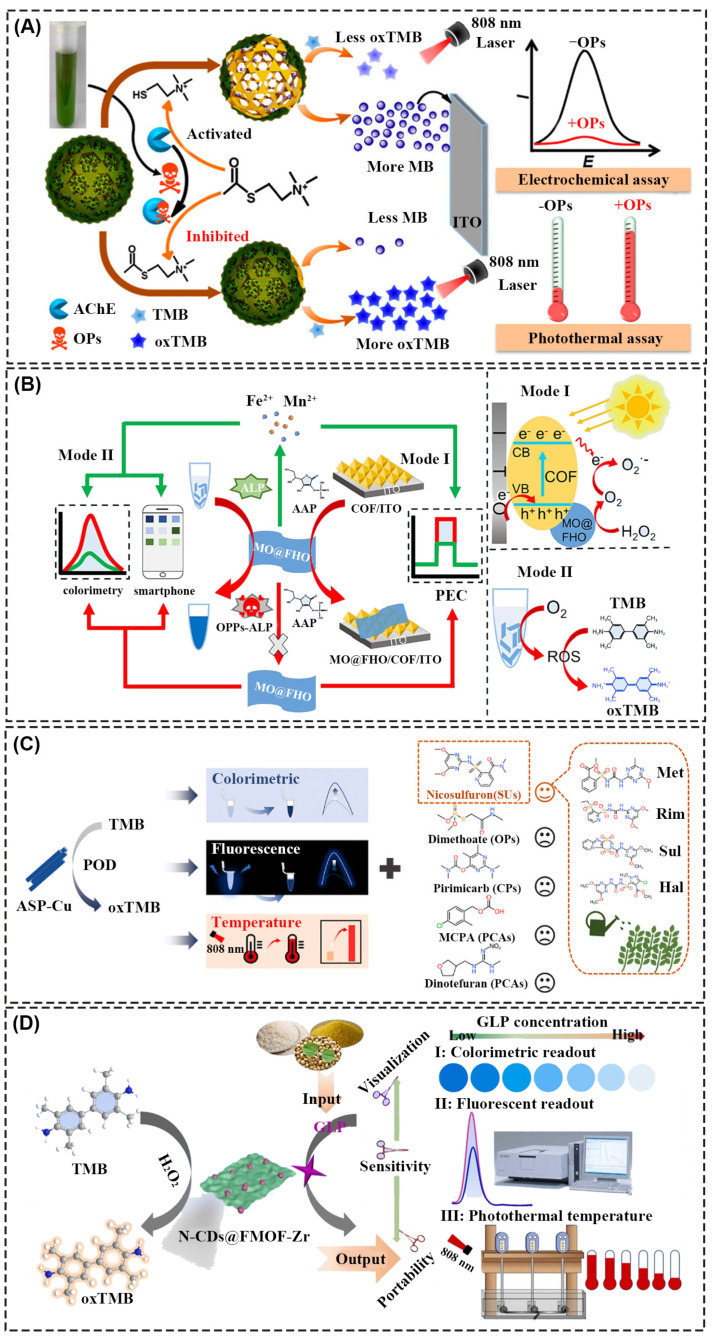
Schematic diagram of other multimodal sensing. (**A**) Electrochemical and photothermal dual-mode sensing based on COF/MB@MnO2. (**B**) Colorimetric and photoelectrochemical dual-mode sensing based on MO@FHO. (**C**) Colorimetric/fluorescence/photothermal tri-mode sensing based on ASP-Cu. (**D**) Colorimetric/fluorescence/photothermal tri-mode sensing based on N-CDs/FMOF-Zr.

For instance, Wen et al. created a homogeneous electrochemical and photothermal dual-mode sensing platform based on a novel COF/methylene blue@MnO_2_ composite nanozyme (COF/MB@MnO_2_) [202], as shown in Figure 10A. Through stimulus response regulation, they detected OPs. Photothermal detection was based on the catalytic oxidation of TMB by the COF/MB@MnO_2_ nanozyme to oxTMB. After the oxTMB was generated by laser excitation at 808 nm, the temperature change was measured using a portable thermometer. Electrochemical detection was based on the MnO_2_ coating layer acting as a “gate control” to prevent the diffusion of methylene blue (MB) into the solution. When there was no pesticide, the AChE activity was normal, and TCh decomposed MnO_2_, releasing MB into the solution. The high diffusion current was detected by DPV. When pesticides were present, they inhibited the AChE activity, reduced TCh, decreased MnO_2_ decomposition, and inhibited the release of MB, ultimately resulting in a decrease in current. The detection limit of chlorpyrifos by this dual-mode sensing platform was 0.108 ng/mL and 0.0632 ng/mL, respectively. This homogeneous electrochemical sensing platform can avoid electrode modification, reduce matrix interference, and combine with photothermal detection to avoid complex sample color interference. It has both laboratory-precise analysis and on-site rapid screening capabilities.

For instance, Zuo et al. constructed a dual-mode biosensor for the detection of organophosphorus pesticides based on an ultrathin FeOOH-coated MnO_2_ nanozyme (MO@FHO) [203], as shown in Figure 10B. The colorimetric detection was based on the oxidation of TMB by MO@FHO to generate blue oxTMB. The photoelectrochemical detection was based on the fact that MO@FHO could act as a p-type semiconductor and generate electron–hole pairs under illumination. Its catalytic activity, similar to catalase, can decompose H_2_O_2_ into O_2_, which can enhance the photocurrent at the cathode. When there is an organophosphorus pesticide present, AA is inhibited from being generated, and MO@FHO is abundant. The photocurrent is enhanced. This dual-mode biosensor, combined with COF photoreactive materials, has a detection limit of 0.017 ng/mL and 0.26 ng/mL for malathion by photoelectrochemical and colorimetric sensing, respectively. It has promising application prospects in biochemical analysis, food safety monitoring, and on-site detection.

To further enhance the validity of sensing platforms, some researchers have proposed a tri-mode sensing method. As shown in Figure 10C, Tian et al. used colorimetric, photothermal, and fluorescence signals based on the ASP-Cu nanozyme reaction as the sensing units for detecting pesticides [218], which enabled the detection of sulfonylurea pesticides in complex environments. For instance, Luo et al. proposed a multifunctional platform with colorimetric, fluorescence, and photothermal signal responses based on an N-CDs/FMOF-Zr nanozyme for the visual [164], sensitive, and portable detection of glyphosate (GLP), as shown in Figure 10D. The colorimetric sensing is based on the peroxidase-like activity of N-CDs/FMOF-Zr in the presence of H_2_O_2_, which catalyzes the generation of blue oxTMB from TMB. GLP inhibits the catalytic reaction by occupying the active site and blocking the Fe^2+^/Fe^3+^ redox cycle, resulting in a decrease in absorbance. The fluorescence sensing is based on the 406 nm fluorescence emitted by TMB under 300 nm excitation. GLP inhibits the peroxidase activity of N-CDs/FMOF-Zr through adsorption and metal coordination, reducing the oxidation of TMB to oxTMB and retaining more unoxidized TMB, leading to enhanced fluorescence. The photothermal sensing is based on the photothermal effect of oxTMB under 808 nm laser irradiation, which inhibits the generation of oxTMB by GLP, resulting in a reduction in temperature increase. This glyphosate detection platform with high sensitivity, multi-mode output, and on-site applicability has detection limits of 0.0131 µg/mL, 0.0015 µg/mL and 0.0115 µg/mL for colorimetry, fluorescence, and photothermal sensing, respectively. The sensing platform adopts mutual verification of the three signals to improve the reliability of the results.

## 4. AI in Nanozymes

With the rapid development of artificial intelligence, it can optimize and analyze multi-dimensional signals of catalytic reactions, detect multiple target substances, and output precise information [219,220,221]. It has demonstrated significant advantages in detecting pesticide residues in food [222]. Currently, the application of AI in nanozymes mainly includes processing sensing signals, using anti-interference algorithms to eliminate the matrix effect, and improving the validity of the results [223,224,225]. However, there are still deficiencies in using AI for the structural design and regulation of active sites of nanozymes.

At present, many researchers have utilized AI to extract features and perform pattern recognition from massive signal data, establishing mappings between pesticide types and sensing signals, and achieving qualitative discrimination of multiple types of pesticides. For instance, Manish et al. prepared an MDC nanozyme modified by metal nanoparticles and carbon nanotubes [226], which has peroxidase-like activity and can catalyze the oxidation of H_2_O_2_ by o-phenylenediamine (OPD) to generate the yellow product oxOPD. Principal component analysis (PCA), a machine learning tool, was used to reduce the dimensionality and perform pattern recognition on the colorimetric response data of the sensor array. By constructing a 3 × 3 sensor array, researchers collected the absorbance data of eight pesticides and generated a matrix containing five repeated experiments. Using the first three principal components extracted by the PCA, namely PC1 = 51.04%, PC2 = 21.54%, and PC3 = 21.36%, they generated a 3D score plot and successfully distinguished all pesticides, even allowing for the non-overlapping separation of structurally similar diethyl cyano phosphonate and deltamethrin. Through AI, this sensor array can achieve precise discrimination of eight pesticides at a concentration of 10 µM, with the linear detection range of carbendazim, deltamethrin, and isoproturon being 1–8 µM, and the detection limits being 10.8, 28.8, and 16.8 nM, respectively. In this method, traditional colorimetric sensing was combined with machine learning through a PCA-driven pattern recognition strategy, overcoming the limitations of the single-receptor “lock-and-key” model and providing an efficient solution for multi-target pesticide detection in complex matrices. Based on regression model analysis of the nonlinear correlation between signal intensity and concentration, pesticide residues can be quantified. Based on the analysis of the nonlinear correlation between signal intensity and concentration using regression models, the pesticide residues can be quantified.

**Figure 11:**
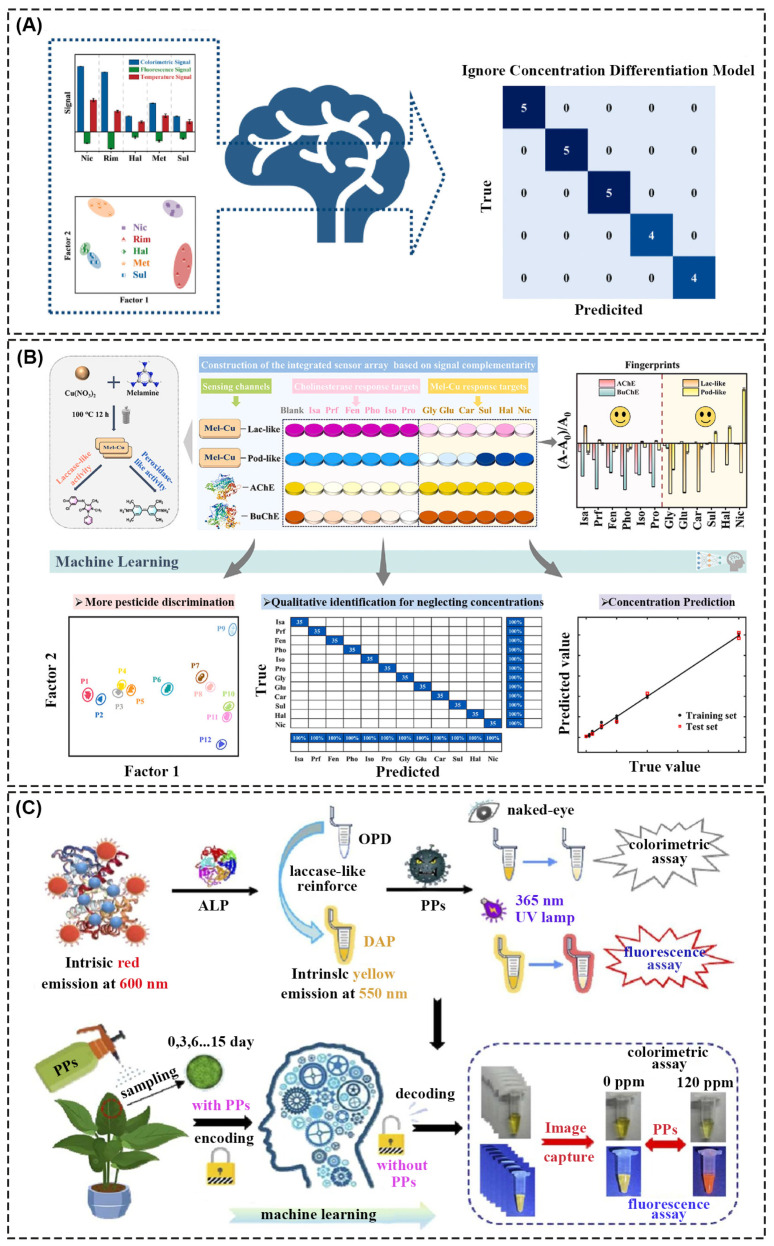
Schematic diagram of pesticide detection based on “AI + nanozymes”. (**A**) Schematic diagram of a multi-signal sensor array based on ASP-Cu assisted by machine learning. (**B**) Schematic diagram of the principle of an integrated sensor array for intelligent pesticide recognition based on Mel-Cu assisted by machine learning. (**C**) Schematic diagram of the principle of colorimetric and fluorescence dual-mode detection based on Cu-ATP@[Ru(bpy)_3_]^2+^ assisted by machine learning.

AI can also eliminate the matrix effect on a single signal through multi-signal fusion and anti-interference algorithms, thereby improving the reliability of detection. For example, Tian et al. combined Cu^2+^ with the ASP ligand under alkaline conditions to synthesize copper-based ASP-Cu nanozymes [218]. Due to the catalytic effect of ASP-Cu in the presence of H_2_O_2_, which oxidizes TMB to generate blue oxTMB, resulting in an absorbance signal at 652 nm, a decrease in fluorescence intensity, and a photothermal heating effect, this team innovatively integrated three signal units of colorimetry, fluorescence, and photothermal, and constructed a multi-dimensional data matrix (Figure 11A). By using the KNN algorithm, a concentration-independent quantitative regression model was constructed. Using the K-nearest neighbor (k = 5) method, the similarity between the test sample and the training set was calculated based on the Euclidean distance, enabling the sensor array to achieve 100% accurate discrimination of five sulfonylurea pesticides (SUs) within the range of 0.1–100 µg/mL, with a detection limit as low as 0.1 µg/mL. When the concentrations of interfering substances such as sugars and metal ions were 25 times higher than those of SUs, the KNN model still maintained a 100% classification accuracy rate, solving the bottleneck of traditional single-signal detection being susceptible to matrix interference and providing a new strategy for the high-throughput screening of sulfonylurea pesticides in complex environments.

The establishment of prediction models through AI can also enable qualitative identification and quantitative determination of multiple targets of pesticides. For instance, Song et al. synthesized a Mel-Cu nanozyme with biomimetic Cu-N sites by using melamine and Cu(NO_3_)_2_ as precursors through a solvothermal method [227]. They constructed a four-channel array sensor by utilizing the dual activity of Mel-Cu and cholinesterase, and combined Random Forest (RF) and Support Vector Machine (SVM) to build a classification–regression joint model for pesticide recognition independent of concentration (Figure 11B). The sensor array successfully distinguished 12 kinds of pesticides, and the linear discriminant analysis and hierarchical clustering analysis showed a classification accuracy of 100%. This research achieved intelligent recognition of multiple types of pesticides by integrating the signal complementarity of nanozymes and natural enzymes and combining it with the machine learning classification–regression joint model. The SVM classification model overcame the interference of concentration, and the SVM regression model achieved precise quantitative analysis, providing a high-precision and high-throughput solution for pesticide residue analysis in complex matrices. Furthermore, by integrating smartphone image recognition with cloud computing, AI can promptly analyze on-site detection data and quickly output visual results, providing a highly sensitive, high-throughput, and portable intelligent solution for food safety supervision.

With the development of AI, the on-site intelligent identification and quantitative detection of pesticides will become more efficient. For instance, Wu et al. synthesized the fluorescent nanozyme Cu-ATP@[Ru(bpy)_3_]^2+^ through coordination self-assembly and electrostatic adsorption strategies [228]. They selectively inhibited ALP activity with pyrethroid pesticides (PPs), reduced DAP production, and caused a decrease in the color signal and an increase in the fluorescence signal, achieving dual-modal detection. At the same time, a smartphone platform based on a WeChat mini-program was developed, capturing dual-modal color and fluorescence images in a dark box. Using the improved YOLOv8 algorithm, combined with convolutional neural networks (CNNs) and ResNet residual modules, Quick-Conv2d was accelerated for feature extraction (Figure 11C). A total of 6400 color/fluorescence images of different concentrations of PPs were prepared and normalized to 224 × 224 pixels for input into the system to build a dataset. Through the Flatten layer and fully connected network, features were summarized, and end-to-end training was used to achieve high-sensitivity prediction of PP concentrations. The detection limit of the color mode was 0.06 ppm, and that of the fluorescence mode was 0.04 ppm. This study achieved high-sensitivity and intelligent on-site detection of PPs by designing dual-modal nanozyme probes and combining machine learning-driven smartphone platforms, providing innovative tools for food safety and environmental health monitoring.

Currently, nanozymes face numerous challenges in the detection of pesticide residues in food such as catalytic activity and stability. The catalytic activity and stability of existing nanozymes need to be enhanced, especially in complex food matrices, where environmental factors such as pH and temperature can impact performance. Selectivity and sensitivity are also challenging aspects. The interactions between different types of pesticides and nanozymes vary significantly, necessitating the development of broad-spectrum detection strategies. The extremely low permissible limits of some pesticides in food demand higher sensitivity from nanozyme sensors. In terms of preparation and cost, some nanozymes require high-temperature pyrolysis or multi-step modification, which are complicated processes with low yields, thus affecting large-scale applications. Moreover, the activity of certain enzymes is reduced when immobilized on nanomaterials, so carrier design needs to be optimized. There are also bottlenecks in experimental applications. The reproducibility of sensors in real samples is insufficient, and standardized sample pretreatment is required. Real-time monitoring takes too long, making it difficult to achieve rapid on-site detection. Future research should focus on developing highly active and stable nanozyme materials. Anti-interference designs should be optimized, and preparation processes should be simplified. The integration of diverse sensing strategies and AI technology will enhance sensitivity, selectivity, and actual applications of nanozymes in food safety monitoring.

Compared with many researchers who have already applied AI to the signal processing and analysis of pesticide detection based on nanozymes, there is still a deficiency in the aspect of designing and constructing nanozymes using AI. AI has shown great potential in the rational design and activity optimization of nanozymes. Through machine learning and deep learning models, researchers can efficiently predict the catalytic type and kinetic parameters of nanozymes and guide the atomic-level regulation of active sites. In the future, by leveraging the advantages of different types of nanozymes, such as the high specific surface area and good biocompatibility of carbon-based nanozymes, the high catalytic activity of noble metal nanoparticles in metal-based nanozymes, the strong redox activity of metal-oxide-based nanozymes, the rich active sites provided by the porous structure of MOF-based nanozymes, the fluorescence-based nanozymes that combine fluorescence signals and catalytic activity, and the versatility of composite material-based nanozymes in complex environments, combined with AI for more purposeful nanozyme construction, innovative solutions will be provided for the analysis and detection of trace pesticides in complex matrices.

## 5. Conclusions

Nanomodules, endowed with their unique biomimetic catalytic activity, high stability, and functional modularity, have emerged as a highly promising technological approach in the field of food pesticide residue detection. Through the innovative design of various substrate materials such as carbon, metal, metal oxides, and MOFs, researchers have successfully developed multiple highly sensitive and selective nanomodule sensing platforms. In this review, we mainly introduce the synthesis methods and catalytic mechanisms of different substrate nanomodules, as well as the application of nanomodule-driven multimodal sensing in practical scenarios. With the rapid development of AI, we also introduce the current application status of “AI + nanozymes”, including using AI to extract features and perform pattern recognition from massive signal data, constructing a self-correcting detection system based on multimodal signal fusion, and achieving qualitative discrimination of multiple types of pesticides. However, research in this field of using AI to construct specific nanomodules for pesticides is currently lacking. In the future, when facing traditional and new chemical pesticides, selectively constructing nanomodules with different substrates and corresponding detection systems will become a research trend. At the same time, by combining smartphone image recognition and cloud computing, AI can analyze on-site detection data in real time and output visual results, providing high-sensitivity and high-throughput intelligent solutions for food safety supervision.

## Figures and Tables

**Table 1:** X-based nanozymes used for pesticide detection.

X-Based	Nanozyme	Detected Pesticides	Linear Range	Detection Limit	Sample	Ref.
Carbon-based	GQDs	Dichlorvos	0.45–45.25 μM	0.778 μM	Water	[93]
	CDs	Paraoxon	0.001–1.0 μg mL^−1^	0.4 ng mL^−1^	Water, Rice, Cabbage	[94]
	SP-BC CH-BC EP-BC	Diafenthiuron, Fomesafen Bensulfuron Methyl Lactofen, Starane	1–500 μM	1 μM	Soil, Water, Seawater Apples, Cucumbers Peaches, Tomatoes, Cabbages	[95]
	NG/NSG/GO	Lactofen, Fomesafen Fluroxypyr-meptyl Bensulfuron-methyl Diafenthiuron	5–500 μM	/	Soil	[96]
	POCNS	Chlorpyrifos	1–200 μg L^−1^	0.31 μg L^−1^	Peach, Apple	[97]
	Cl_x_-pNC	Glyphosate	0.25–7 μM	0.07 μM	Apple	[98]
	Mn@NC	Phoxim	0.05–5000 ng mL^−1^	0.011 ng mL^−1^	Fruit, Vegetable	[99]
	Cu@NC	Thiophanate-methyl	0.2–15 µg mL^−1^	0.04 µg mL^−1^	Water, Soil	[100]
	SA-CoN_3_	Glyphosate	0–40 μM	0.66 μM	Water, Apples, Pears Peaches, Grapes	[101]
	FeAC/FeSA-NC	OPs	0.005–50 ng mL^−1^	1.9 pg mL^−1^	Water	[102]
	Cu-N-C	Paraoxon-ethyl	1–300 ng mL^−1^	0.60 ng mL^−1^	Water	[103]
Metal-based	GMP-Cu BPY-Cu MIZ-Cu ASP-Cu	Glyphosate, Glufosinate Phosmet, Malathion Fenitrothion, Isocarbophos	0.1–20 μg mL^−1^	10 µg mL^−1^	Apple, Pear, Celery Nectarine, Tomato Cabbage	[104]
	Pt NPs	Dursban, Glyphosate, Malathion Dimethoate, 3-ketocarbofuran	0.5–9 μg mL^−1^	0.15 µg mL^−1^	Apple skin	[105]
	Ir(III)/GO	Pirimicarb	10–300 nM	2.81 nM	Pakchoi	[106]
	N-Pdene	Glyphosate	0.1–50 µM	0.27 µM	Fruits	[107]
	Au@Pt	Parathion, Triazophos Chlorpyrifos	/	1.47 ng kg^−1^	Cabbage, Apple Pear, Rice	[108]
	PtPd NPs	Ethyl paraoxon	0.05–6.4 nM	0.025 nM	Human plasma	[109]
	Pt-Ni NPs	Chlorpyrifos	0.005–3.0 μg mL^−1^	1.2 ng mL^−1^	Wine	[110]
Metal-oxide-based	2D-VONz	Glyphosate	0.1–6 μM	0.026 μM	Water	[111]
	MnNS	Paraoxon	0.1–20 ng mL^−1^	0.025 ng mL^−1^	Pakchoi	[112]
	Ce_2_O_2_CN_2_/NC	Paraoxon	0.1–144 μM	0.135 μM	Water	[113]
	Ag_2_O	Phoxim, Dimethoate, Chlorpyrifos, Triazophos Parathion-methyl Trichlorphon	10–500 ng mL^−1^	10 ng mL^−1^	Vegetable melon	[114]
MOF-based	66-IS-Zn	Glyphosate, Phoxim, Diazinon Glufosinate, Profenofos Methyl Parathion	30–120 μg mL^−1^	30 μg mL^−1^	Cherry tomatoes	[115]
	C_60_@MOF-545-Fe	Glyphosate, Omethoate Paraoxon	0.5–800 ng·mL^−1^	0.16 ng·mL^−1^	Soybean, Apple Rice	[116]
	NH_2_-CuBDC	Chlorpyrifos	1.8–180 ng·mL^−1^	1.57 ng mL^−1^	Apple	[117]
	His-MIL-101(Fe)-X	Diafenthiuron, Lactofen Fluoroxypyr-meptyl Bensulfuron Methyl Fomesafen	2–100 μM	2 μM	Soil Water Apple	[118]
Fluorescence-based	Cu-BDC-NH_2_	Malathion, Glyphosate Dimethoate, Phosmet m-tolylmethylcarbamate Dicofol, Fenvalerate Pirimicarb, Etoxazole Metsulfuron-methyl	1–100 μg·mL^−1^	1 μg·mL^−1^	Chilli, Pear Celery, Tomato Cherry, Nectarine	[119]
	Fe-CDs/MOF-808 Fe-CDs@MOF-808	Paraoxon Parathion	0.001–360 μM	0.3 nM	Water Pakchoi	[120]
	aZIF-8@CuNCs	Chlorpyrifos	0–50 μg·mL^−1^	0.43 μg·mL^−1^	White Radishes Cherry Tomatoes, Planting Soil	[121]
	NH-MnCu NFs	2,4-dichlorophenol Methyl parathion	1–125 ng·mL^−1^	0.36 ng·mL^−1^	Apple, Leek Water	[122]
	GQD@Tb/GMP ICP	Parathion	0.1–100 ng·mL^−1^	0.037 ng·mL^−1^	/	[123]
Other X-based	CuNFs-Apt-AChE	Metolcarb	1–1000 ng mL^−1^	0.19 ng mL^−1^	Water, Apple Juice	[124]
	AuNCs@MnO_2_	Paraoxon	5–500 ng mL^−1^	5.0 ng mL^−1^	Water, Milk, Orange Juice	[125]
	BSPOTPE-SiO-MnO_2_	Paraoxon	1–100 μg L^−1^	1 μg L^−1^	Water	[126]
	Asp-Cu	Glyphosate, Phosmet Isocarbophos, Carbaryl Pentachloronitrobenzene Metsulfuron-methyl, Etoxazole 2-methyl-4-chlorophenoxyac-etic Acid	0.1–100 μg mL^−1^	0.1 μg mL^−1^	Broccoli Cabbage Tomato	[127]
	AChE-MnO_2_@HPH	Fenthion	4–400 ng mL^−1^	0.63 ng mL^−1^	Rice, Wheat	[128]
	Fe_3_O_4_@Cu	Glyphosate	0.05–0.17 μg mL^−1^	0.019 µg mL^−1^	Peas, Oranges, Water	[129]

## Data Availability

No new data were created or analyzed in this study. Data sharing is not applicable to this article.

## Abbreviations

The following abbreviations are used in this manuscript:

2D-VONz	Two-dimensional V_2_O_5_ nanosheets
2D-ZHM	Two-dimensional hemin-bridged MOF nanozyme
AA	Ascorbic acid
AAP	Ascorbic acid-2-phosphate
ACC-HNFs	All-in-one enzyme–inorganic hybrid nanoflowers
AChE	Acetylcholinesterase
ACP	Acid phosphatase
ACQ	Aggregation-induced quenching
ALP	Alkaline phosphatase
Apt	Aptamers
APTES	3-aminopropyl triethoxysilane
AR	Amplex Red
Asp	L-aspartic acid
ASP-Cu	L-aspartic acid–copper
AuNCs	Gold nanoclusters
aZIF-8	Amorphous zeolite imidazole framework-8
BNPP	Bis(4-nitrophenyl) phosphate
BPY-Cu	4,4′-bipyridine–copper
BSA	Bovine serum albumin
C_60_	Fullerene
Ce (OAc)_3_	Cerium acetate
CH-BC	Chlorella biochar
CHOx	Choline oxidase
Cl_x_-pNC	Cl and O dual-doped nitrogen-doped porous carbon nanoezymes
CNN	Convolutional neural network
COF/MB@MnO_2_	A novel COF/methylene blue@MnO_2_ composite nanozyme
CPNs	Copper peroxide nanodots
CPNs(IV)	Cerium-based coordination polymer nanoparticles
Cu@NC	Copper-doped carbon-based nanozyme
CuCl	Cuprous chloride
CuNCs	Glutathione-capped copper nanoclusters
CuNFs	DNA-Cu nanoflowers
DA	Dopamine
DAP	2,3-diaminophenazine
DFQ	3-(1,2-dihydroxyethyl) furan [3,4-b] quinoline-1(3H)
DICY	Dimethylglyoxime
EP-BC	Enteromorpha biochar
FDP	Fluorescein diphosphate
FMOF-Zr	Zr-based ferrocene metal–organic framework
FRET	Förster resonance energy transfer
GMP	Guanosine monophosphate
GMP-Cu	Guanosine 5′-monophosphate–copper
GO	Graphene oxide
GQDs	Graphene quantum dots
H_2_BDC	2,5-dihydroxybenzoic acid
h-BN QDs	Hydroxyl-functionalized hexagonal boron nitride quantum dots
HCA	Hierarchical clustering analysis
His	Histidine
ICP	Infinite coordination polymer
IFE	Internal filter effect
K_2_HPO_4_	Dipotassium hydrogen phosphate
LAC	Laccase-like
LDA	Linear discriminant analysis
LOD	Limit of detection
MB	Methylene blue
MIECL	Molecular imprinting electrochemiluminescence
MIZ-Cu	2-methylimidazole–copper
Mn@NC	Manganese–nitrogen-co-doped carbon-based nanoenzymes
MnCu NFs	Manganese–copper nanoflowers
MnNS	MnO_2_ nanosheets
MO@FHO	Ultrathin FeOOH-coated MnO_2_ nanozyme
MOF	Metal–organic framework
MRLs	Maximum residue limits
N-CDs	Nitrogen-doped carbon dots
NG	Nitrogen-doped graphene
NH_2_-BDC	2-aminoterephthalic acid
N-Pdene	Pd metalene nanoenzyme
NPs	Nanoparticles
NSG	Nitrogen- and sulfur-co-doped graphene
OPD	O-phenylenediamine
OPH	Organophosphorus hydrolase
OPs	Organophosphorus pesticides
PCA	Principal component analysis
PCN-224-Mn	Manganese-modified porphyrin metal–organic framework
PDA	Polydopamine
PEG	Polyethylene glycol
POCNS	Phosphorus–oxygen-co-doped carbon nanosheet
POD	Peroxidase-like
POMS	Peroxymonosulfate
PPs	Pyrethroid pesticides
Pt NPs	Platinum-based nanoparticles
Pt-Ni NPs	Platinum–nickel bimetallic nanoparticles
PVP	Polyvinylpyrrolidone
RASFF	Rapid Alert System for Food and Feed
RF	Random Forest
ROS	Reactive oxygen species
SA-CoN_3_	Cobalt single-atom nanoenzymes with unsaturated coordination structure
SDBS	Sodium dodecyl benzene sulfonate
SOD	Superoxide dismutase-like
SP-BC	Spirulina biochar
SPCE	Screen-printed carbon electrode
SPE	Solid-Phase Extraction
SUs	Sulfonylurea pesticides
SVM	Support Vector Machine
TEOS	Tetraethyl orthosilicate
TM	Thiophanate-methyl
TMB	3,3′,5,5′-tetramethylbenzidine
TPPO	Triphenylphosphine oxide
ZIFs	Zeolite imidazolate framework
